# Wiskott Aldrich Syndrome: A Multi-Institutional Experience From India

**DOI:** 10.3389/fimmu.2021.627651

**Published:** 2021-04-16

**Authors:** Deepti Suri, Rashmi Rikhi, Ankur K. Jindal, Amit Rawat, Murugan Sudhakar, Pandiarajan Vignesh, Anju Gupta, Anit Kaur, Jyoti Sharma, Jasmina Ahluwalia, Prateek Bhatia, Alka Khadwal, Revathi Raj, Ramya Uppuluri, Mukesh Desai, Prasad Taur, Ambreen A. Pandrowala, Vijaya Gowri, Manisha R. Madkaikar, Harsha Prasada Lashkari, Sagar Bhattad, Harish Kumar, Sanjeev Verma, Kohsuke Imai, Shigeaki Nonoyama, Osamu Ohara, Koon W. Chan, Pamela P. Lee, Yu Lung Lau, Surjit Singh

**Affiliations:** ^1^ Department of Pediatrics, Post Graduate Institute of Medical Education and Research (PGIMER), Chandigarh, India; ^2^ Department of Haematology, Post Graduate Institute of Medical Education and Research (PGIMER), Chandigarh, India; ^3^ Department of Internal Medicine, Post Graduate Institute of Medical Education and Research (PGIMER), Chandigarh, India; ^4^ Department of Paediatric Haematology and Oncology, Apollo Speciality Hospitals, Chennai, India; ^5^ Division of Immunology, Bai Jerbai Wadia Hospital for Children, Mumbai, India; ^6^ Department of Paediatric Immunology and Leukocyte Biology, National Institute of Immunohematology, Mumbai, India; ^7^ Department of Pediatrics, Kasturba Medical College, Mangalore, Manipal Academy of Higher Education, Manipal, India; ^8^ Pediatric Immunology and Rheumatology, Aster CMI Hospital, Bengaluru, India; ^9^ Department of King George Medical University, Lucknow, India; ^10^ Department of Pediatrics, Tokyo Medical and Dental University (TMDU), Tokyo, Japan; ^11^ Department of Pediatrics, National Defense Medical College, Saitama, Japan; ^12^ Department of Applied Genomics, Kazusa DNA Research Institute, Kisarazu, Chiba, Japan; ^13^ Department of Pediatrics and Adolescent Medicine, Li Ka Shing Faculty of Medicine, Queen Mary Hospital, The University of Hong Kong, Hong Kong, Hong Kong

**Keywords:** thrombocytopenia, X-linked thrombocytopenia, microplatelets, hematopoetic stem cell transplant, WASP, autoimmunity, bleeding, malignancy

## Abstract

**Background:**

Wiskott Aldrich syndrome (WAS) is characterized by bleeding manifestations, recurrent infections, eczema, autoimmunity, and malignancy. Over the last decade, improved awareness and better in-house diagnostic facilities at several centers in India has resulted in increased recognition of WAS. This study reports collated data across major primary immunodeficiency diseases (PID) centers in India that are involved in care of children with WAS and highlights the varied clinical presentations, genetic profile, and outcomes of patients in India.

**Methods:**

Request to share data was sent to multiple centers in India that are involved in care and management of patients with PID. Six centers provided requisite data that were compiled and analyzed.

**Results:**

In this multi-institutional cohort, clinical details of 108 patients who had a provisional diagnosis of WAS were received. Of these, 95 patients with ‘definite WAS’ were included Fourteen patients were classified as XLT and 81 patients as WAS. Median age at onset of symptoms of patients was 3 months (IQR 1.6, 6.0 months) and median age at diagnosis was 12 months (IQR 6,48 months). Clinical profile included bleeding episodes (92.6%), infections (84.2%), eczema (78.9%), various autoimmune manifestations (40%), and malignancy (2.1%). DNA analysis revealed 47 variants in 67 cases. Nonsense and missense variants were the most common (28.4% each), followed by small deletions (19.4%), and splice site defects (16.4%). We also report 24 novel variants, most of these being frameshift and nonsense mutations resulting in premature termination of protein synthesis. Prophylactic intravenous immunoglobulin (IVIg) was initiated in 52 patients (54.7%). Hematopoietic stem cell transplantation (HSCT) was carried out in 25 patients (26.3%). Of those transplanted, disease-free survival was seen in 15 patients (60%). Transplant related mortality was 36%. Outcome details were available for 89 patients. Of these, 37% had died till the time of this analysis. Median duration of follow-up was 36 months (range 2 weeks- 12 years; IQR 16.2 months- 70 months).

**Conclusions:**

We report the first nationwide cohort of patients with WAS from India. Bleeding episodes and infections are common manifestations. Mortality continues to be high as curative therapy is not accessible to most of our patients.

## Introduction

Wiskott–Aldrich syndrome (WAS; OMIM#301000) is an X- linked immune deficiency disorder with an estimated incidence of 3.7- 4.1 per 1 million live births, and is characterized by micro- thrombocytopenia, eczema, combined immunodeficiency, and increased risk for autoimmunity, and malignancy (1–4).

This syndrome is caused by mutations in *WAS* gene that contains 12 exons and is located on short arm of X chromosome (Xp11.23) (5). *WAS* gene encodes Wiskott Aldrich syndrome protein (WASp), which is a 502-amino acid protein, and a key molecule for actin cytoskeleton polymerization (6–9). WASp is expressed by all hematopoietic cells (10) and has essential cellular functions like formation of immunological synapses (11–15), release of secretory granules (16, 17), phagocytosis (18, 19), cellular migration (20, 21), and motility (22).

Review of literature revealed occasional case reports with limited information on genetic abnormalities in WAS from India (23–32). We published a small series of eight patients in 2012 highlighting that under-reporting was mainly due to lack of awareness amongst medical fraternity and nonavailability of diagnostic and therapeutic facilities (23). In 2011, a dedicated society for PID (Indian Society for Primary Immune Deficiency, ISPID) was founded. ISPID has been working toward increasing awareness regarding PIDs and establishment of diagnostic support and research centers in the country. ISPID with the support of Foundation of Primary Immunodeficiency Diseases (FPID), USA organized national, international level conferences for sensitization, and further research in field of PIDs. The Indian Council of Medical Research (ICMR) helped set up the Centre for Advanced Research (CAR) facility in PIDs at PGIMER, Chandigarh in 2015 and subsequently at the National Institute of Immunohaematology (NIIH), Mumbai in 2017. There seems to be a paradigm shift in number of patients diagnosed with PID in India after these CAR facilities were started (33). With improved awareness and advent and availability of better genetic diagnostic tests, patients with WAS and other PIDs are now being diagnosed at several centers.

This study reports data across major centers in India that are involved in care of children with PID and highlights the clinical manifestations and genetic profiles. It also emphasizes the difficulties likely to be encountered in management of these patients in context of a developing country.

## Patients and Methods

All members of ISPID were also contacted *via* email to share data of patients with WAS on a predesigned excel sheet by the lead author (DS). Different centers supported by the FPID, USA, and other institutions involved in care of patients with PID across India were also contacted. Data including demographics, prominent clinical manifestations, laboratory investigations, genetic results, treatment regimens and long-term outcomes were collated on an excel sheet. (appendix1) Participating centers included Postgraduate Institute of Medical Education and Research (PGIMER), Chandigarh, North India (60 patients); Apollo Hospitals, Chennai, South India (19 patients); Bai Jerbai Wadia Hospital for Children, Mumbai, West India (16 patients); Aster CMI Hospital, Bengaluru, South India (5 patients); Kasturba Medical College, Manipal Academy of Higher Education, South India (4 patients); and King George’s Medical University, Lucknow, North India (1 patient). Ethical approval was obtained from the Institute’s Ethics Committee (INT/IEC/1216).

European Society of Immunodeficiencies (ESID) definitions were used to categorize patients for this analysis (34). The term ‘definite WAS’ was used when there was congenital thrombocytopenia and small platelets and at least one of the following: 1. Mutation in WAS gene; 2. Absent WASp mRNA on northern blot analysis of lymphocytes; 3. Absent WASp protein in lymphocytes; 4. Maternal cousins, uncles or nephews with small platelets and thrombocytopenia). ‘Probable WAS’ was diagnosed, if patients had congenital thrombocytopenia, small platelets and at least one of the following: 1. Eczema; 2. Abnormal antibody response to polysaccharide antigens; 3. Recurrent bacterial or viral infections; 4. Autoimmune diseases; 5. Lymphoma, leukemia, or brain tumor (34).

The WAS severity scoring system was used to differentiate between patients with mild and severe clinical phenotypes (35). Patients with ‘mild’ WAS phenotype or XLT had thrombocytopenia with absent or isolated and intermittent eczematous lesions and absence of recurrent or chronic infections (WAS score 1 or 2). Patients with classic WAS had extensive and difficult-to-treat eczema or had recurrent infections requiring frequent antimicrobial agents (score 3 or 4). Patients who developed autoimmunity or malignancy received a score of 5 (35). The collated data include demographic profile, clinical presentation, investigation details including platelet counts, mean platelet volume (MPV), and serum immunoglobulins (IgG, IgA, IgM, IgE). MPV value between 7.5 -11 fL was considered normal, and microplatelets were defined if MPV was < 7.5 fL.

DNA analysis of WAS was carried out to confirm the diagnosis in patients with clinical suspicion of WAS and in those who had low MPV or reduced WASp expression, or suggestive X-linked family history.

PGIMER, Chandigarh was designated as a Centre for Advanced Research for Primary Immunodeficiency Disorders by ICMR in 2015 and was soon followed by NIIH, Mumbai. Facilities for flow cytometry and genetic studies are available at both these centers. Most patients diagnosed at Bai Jerbai Wadia Hospital for Children (BJWHC), Mumbai, underwent investigations in collaboration with NIIH, Mumbai. At most other centers, flow cytometry and NGS based molecular test are outsourced to commercial laboratories. Hematopoietic Stem Cell Transplant (HSCT) was carried out at PGIMER, BJWHC, Apollo Hospitals and Aster CMI Hospital, Bengaluru.

### Laboratory Investigations at PGIMER, Chandigarh

Platelet count and size (MPV) was estimated by automated analyzers (COULTER^®^ HmX AL Analyzer, Beckman Coulter, United States or COULTER^®^ LH780 Hematology Analyzer, Beckham Coulter, United States) standardized for MPV estimation. A dedicated peripheral smear examination to look for platelet morphology was also carried out by an experienced hematologist as automated analyzers can dismiss small thrombocytes as debris leading to erroneously low platelet count and high MPV. Serum IgG, IgM and IgA were estimated by nephelometer (MININeph, semiautomated nephelometer, The Binding Site, United Kingdom) while serum IgE was estimated by enzyme immunoassay.

### WASp Quantification by Flow Cytometry

Intracellular staining of WASp using phycoerythrin (PE)-labeled anti human WASp antibody (sc-13139PE (clone: B-9), Santa Cruz Biotechnology, United States), was carried out on non-erythroid blood cells in peripheral blood. Cells were gated using side scatter vs CD45 labeled with fluorescein isothiocyanate (FITC) (A07782; Beckman Coulter Life Sciences, United States). Cells were acquired on an in-house flow cytometer (Navios™ EX System, Beckman Coulter, United States). Median fluorescence intensity (MFI) and Stain Index (SI) in stimulated and unstimulated samples were calculated.

### DNA Amplification and Sequencing

Prior to 2015, DNA analysis for WAS was carried out in collaboration with overseas centers, which included: Service d’Hématologie, d’Immunologie et de Cytogénétique, Hôpital de Bicêtre, Le Kremlin-Bicêtre, France; National Defense Medical College, Saitama, Japan; and Department of Paediatrics & Adolescent Medicine, The University of Hong Kong, Hong Kong.

After 2015, DNA analysis was performed in-house in Pediatric Immunology laboratory, Advanced Pediatrics Centre by Sanger sequencing using specific primers. After obtaining informed consent from parents or caregivers, genomic DNA was isolated from peripheral blood samples using Qiagen kits (QIAMP DNA Blood Mini Kit, 51106, Qiagen Ltd., United States). All 12 exons of *WAS* gene were amplified by polymerase chain reaction (PCR) using 9 sets of specific primers. Primer sequences and PCR conditions are attached as Appendix1.

Direct sequencing was performed at Central Sophisticated Instrument Centre on a genetic analyzer (Applied Biosystems^®^ 3500 Series Genetic Analyser, Thermo Fisher Scientific, United States). Sequences were aligned with known *WAS* gene sequence (NCBI reference sequence: NG_007877.1). Sequence variations were described according to reference sequences (Ensembl ID: ENST00000376701.5), and cDNA nucleotides were counted from the first ATG translation initiation codon. *WAS* gene variants were confirmed with Ensembl, Genome Aggregation Database (gnomAD) and Human Gene Mutation Database (HGMD).

Since 2018, we have started performing targeted next generation sequencing (NGS) for PID patients using a gene panel comprising of 44 genes including the WAS gene on IonS5 platform, (Ion torrent S5, Thermo Fisher Scientific, United States). However, variants in regulatory regions and the polyadenylation sites of the *WAS* gene were not covered by the panel that we have been using.

### Laboratory Investigations at Other Centers

At other centers, WASp expression could only be carried out in some patients due to non-availability of this specialized flow cytometric test. Genetic tests were performed based on clinical suspicion. NGS using a targeted gene panel was also performed by most centers through commercial laboratories (MedGenome Laboratories Pvt Ltd). Illumina platform was used for sequencing in the commercial laboratories with a coverage of >80X. Sanger sequencing was used to confirm the variants obtained by NGS.

### Management and Treatment

Appropriate antimicrobial therapy was initiated to treat breakthrough infections based on clinical manifestations, microbiological susceptibility, and clinical judgment of the treating physician. Patients with classic WAS were initiated on cotrimoxazole prophylaxis and monthly intravenous immunoglobulin (IVIg) therapy, while patients with XLT phenotype were managed symptomatically. Most patients with XLT were not initiated on any prophylactic therapy. Indications for replacement IVIg were: WAS score 3-5, frequent infections despite antimicrobial prophylaxis, patients awaiting HSCT. Corticosteroids were used for treatment of skin vasculitis and autoimmune hemolytic anemia (AIHA), and malignancies were treated using standard chemotherapeutic protocols. HSCT was carried out in patients for whom a suitable donor could be arranged, at a center of their choice.

### Statistical Analysis

Patient demographics, disease and treatment related variables were summarized using descriptive statistics such as medians and ranges for continuous variables and counts and percentages for categorical variables. Mann Whitney U test was used to compare continuous variables between groups. Categorical variables were compared by using the x^2^ test or Fisher’s exact test wherever needed. The Kaplan-Meier method was used to estimate probability of overall survival (OS). Death from any cause was considered as an event, and surviving patients were censored at last follow-up. Log-rank test was used to compare overall survival probability between different groups of patients. All p-values were 2-sided, and a p value of <0.05 was considered significant.

## Results

**Figure 1:**
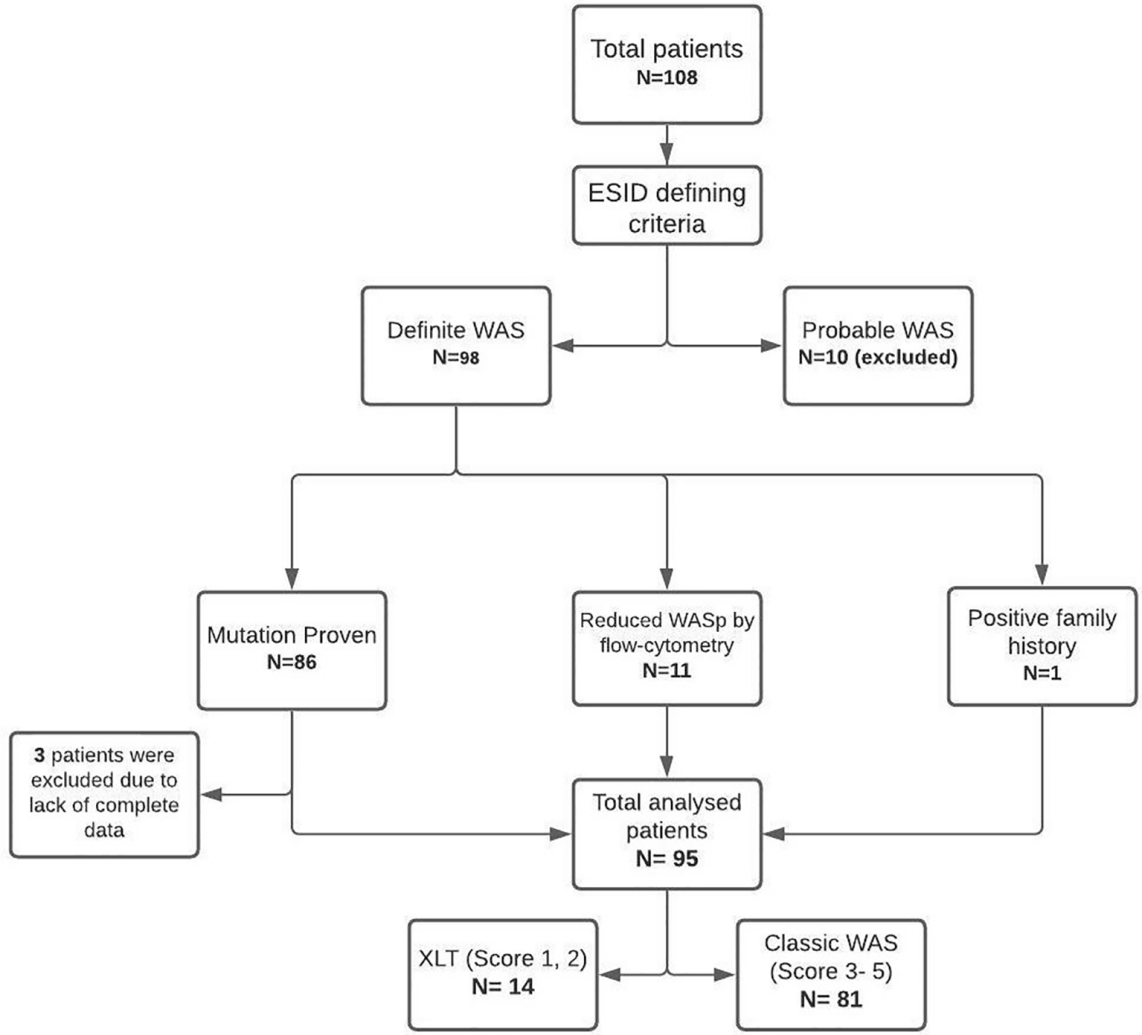
STROBE diagram of collated data, stratification and inclusion of study subjects.

Data of 108 patients with WAS, collated from 6 centers, were included and analyzed. Ninety-eight patients had ‘definite WAS’, and 10 patients had ‘probable WAS’. Patients with ‘probable WAS’ and 3 patients with ‘definite WAS’ with insufficient clinical and demographic details were excluded from analysis. The study thus included 95 patients with WAS for further analysis (**Figure 1**). Complete variant details were available for 67 of 86 patients who had been confirmed by genetic tests. The cohort included 14 patients of XLT (WAS score 1- 7 children; score 2- 7 children), while remaining 81 children had classic WAS (WAS score 3- 29 patients; score 4- 13 patients; and score 5- 39 patients).

Median age at onset of symptoms was 3 months (range birth -14 years; interquartile range, (IQR) 1. - 6 months) while median age at diagnosis was 12 months (range 2 month – 32 years; IQR 6- 48 months) (**Table 1**). Median age of alive children at the time of analysis was 86.5 months (IQR: 52- 168 months, range: 10- 418 months). Median delay in diagnosis was 8 months (range 1 month – 16.5 years; IQR 3- 30.2 months) and median follow-up was 36 months (range 2 weeks- 12 years; IQR 16.2 months- 70 months). Family history of WAS or X-linked inheritance was present in 29 patients (30.5%). Details of median age at onset of symptoms and age at diagnosis in patients with XLT and WAS have been summarized in **Table 1**. Patients with classic WAS earlier onset of symptoms, were younger at time of diagnosis and had less delays in establishment of diagnosis in comparison to patients with XLT (p values were 0.044, 0.019, and 0. 021 respectively) (**Table 1**).

**Table 1:** Baseline characteristics of the included patients.

Parameter	Entire cohort (n=95)	XLT (n=14)	WAS (n=81)	p value*
Median age at onset of symptoms (IQR, months)^a^	3 months (1.6- 6)	6 months (2.5- 48)	3 months (1.25- 6)	0.044
Median age at diagnosis (IQR, months)^a^	12 months (6- 48)	51 months (10 – 102)	12 months (5.5- 36)	0.019
Median delay in diagnosis (IQR, months)^a^	8 months (3- 30.2)	31 months (6.5- 69)	7 months (3- 24)	0.021
Median follow-up (IQR, months)^a^	36 months (16.2- 70)	40 months (26.5- 75)	36 months (12.2- 70)	0.496
Median value of MPV (IQR)^a^	5.6 fL (5- 7)	5.4 fL (4- 6.9)	5.6 fL (5- 7)	0.702
Median platelet count (IQR)^a^	20x10^9^/L (12- 33x10^9^/L)	22x10^9^/L (5- 43 x10^9^/L)	20x10^9^/L (12- 31x10^9^/L)	0.943
Median WASp SI (IQR)^a^	0.33 (0.24- 0.60)	0.36 (0.13- 0.49)	0.32 (0.25- 0.61)	0.259
Number of patients with mortality^b^	33	1	32	0.028

IQR, Interquartile range.

a Mann-Whitney U test.

b Fisher’s exact test or chi-squared test.

*p value comparison between XLT and WAS, < 0.05 is significant.

### Clinical Manifestations at First Presentation

Most children with WAS (71, 74.7%) had bleeding manifestations at time of presentation, commonest being gastrointestinal bleeding in form of blood-stained stools (47, 49.4%), followed by eczema and infections **(** **Table 2** **)**. Clinical triad of bleeding, eczema, and infections was seen in 15 patients (15.7%) at time of presentation, though complete triad evolved over time in 70 patients (73.6%). Three patients had presented with autoimmune manifestations (AIHA 2 patients; skin vasculitis 1 patient) and were later diagnosed to have WAS (**Table 2**).

**Table 2:** Clinical manifestations at the time of presentation in the cohort.

Presenting Manifestations	No. of patients (n=95)	%
**Bleeding**	71	74.7
Blood in stools	47	49.4
Skin bleeds	19	20
Skin and mucosal bleed	5	5.3
Epistaxis	5	5.3
Hematuria	4	4.2
Hematemesis	2	2.1
Bleeding from >1 site	11	11.5
**Eczema**	44	46.3
**Infections**	41	43.1
Pneumonia	19	20
Otitis media	5	5.3
CMV infection	2	2.1
Chronic diarrhea	3	3.1
Infection at >1 site	6	6.3
**Autoimmune Manifestations**	3	3.1
AIHA	2	2.1
Skin vasculitis	1	1.0

CMV, Cytomegalovirus; AIHA, autoimmune hemolytic anemia.

Data of provisional diagnosis proffered to patients before referral was available in 42 patients. Eight of these 42 patients (19%) were initially diagnosed and managed as ITP. Diagnosis of WAS (WAS: 5 patients; XLT: 3 patients) was later suspected on based family history (2 patients), non-responsiveness to treatment (6 patients), development of infections (2 patients) or eczema (1patient).

### Clinical Manifestations on Follow-Up

Spectrum of clinical features of our cohort over the follow- up period was summarized in the **Table 3**.

**Table 3:** Clinical manifestations encountered in patients over 217 patient years of follow-up (n=95).

Clinical Manifestations	No of patients (n=95)	%
**Bleeding**	88	92.6
Blood in stools	67	70.5
Skin bleed	54	56.8
Bleeding >1 site	45	47.3
Epistaxis	11	11.5
Hematuria	6	6.3
Hematemesis	2	2.1
Hemoptysis	1	1
Ear canal bleed	1	1
Intracranial bleed	5	5.2
**Infections**	80	84.2
Bacterial		
Pneumonia	52	54.7
Otitis Media	34	35.7
Septicemia	4	4.2
Meningitis	4	4.2
Others*	9	9.4
Viral		
*Cytomegalovirus* infection	7	7.3
*Molluscum contagiosum*	5	5.2
*Epstein Barr virus* infection	2	2.1
Varicella	4	4.2
**Eczema**	75	78.9
**Atopy^#^**	4	4.2
**Autoimmune Manifestations**	38	40
Autoimmune hemolytic anemia	9	9.5
Positive direct Coombs test	9	9.5
Antinuclear antibodies	9	9.5
Skin vasculitis	9	9.5
Takayasu arteritis	1	1.1
Guillain Barre syndrome	1	1.1
Autoimmune lymphoproliferative Syndrome- Like	1	1.1
Primary sclerosing cholangitis	1	1.1
>1 autoimmune manifestation	14	14.7
Others**^##^**	5	5.2
**Neoplasms**	2	2.1

*Chronic infective diarrhea (3); Suppurative lymphadenitis (3); Gluteal abscess (1); Septic arthritis (1).

^#^Asthma (2); Lactose intolerance (1); Cow milk protein allergy (1).

^##^Hypothyroidism (2); Perinicious anemia (1); Alopecia (1); Nephritis (1).

#### Bleeding Manifestations

Bleeding manifestations were encountered in 88 children (92.6%) with blood-stained stools (67; 70.5%) and skin bleeds (54; 56.8%) being the common symptoms (**Table 3**). Other manifestations included epistaxis (11 patients), hematuria (6 patients), hematemesis (2 patients), hemoptysis (1patient) and bleeding from auditory canal (1 patient). Intracranial bleeding was observed in 5 patients (5.3%). Bleeding from multiple sites (more than 1) was found in 45 patients (47.3%) during follow-up.

#### Infections

Eighty patients (84.2%) had evidence of infections with pneumonia being the most common (52, 54.7%), followed by otitis media in 34 patients (35.7%). Septicemia, meningitis, septic arthritis, and skin and soft tissue abscesses were other infections encountered in these patients. Detailed microbiological profile of these infections was not available, as many patients had been treated at peripheral healthcare facilities and records were not available.

*Cytomegalovirus* (CMV) infection was documented in 7 (7.3%) patients. Four amongst these had been treated as congenital CMV infection. Immunological investigations (CD3^+^, CD 19^+^, and CD56^+^ cell populations and serum immunoglobulins) were non-contributory. Diagnosis of WAS was suspected much later when they continued to have persistent thrombocytopenia despite treatment, suggestive family history and/or eczema. One patient developed skin vasculitis in at 11 months of age and further work-up led to diagnosis of WAS.

*Molluscum contagiosum* (MC) *virus* infection was seen in 5 patients - infection was difficult to eradicate in one of these. Varicella infection was seen in 4, of which 2 had severe hemorrhagic manifestations. *Epstein Barr* virus infection was recorded in 2 patients.

#### Eczema and Atopy

Eczema was observed in 75 patients (78.9%). Other allergic manifestations included bronchial asthma (2 patients), lactose intolerance (1 patient) and cow milk protein allergy (1 patient).

#### Autoimmunity

Autoimmune manifestations were observed in 38 patients (40%). AIHA was the commonest manifestation with Direct Coombs test (DCT) being positive in 9 children (9.5%). Two children, aged 6 months and 3.5 years, presented with AIHA and it was only due to high index of suspicion based on other clinical findings, that the diagnosis of WAS could be established. Skin vasculitis was recorded in 9 children (9.5%). Diagnosis of WAS was made because of persistent micro-thrombocytopenia. Incidental antinuclear antibodies (ANA) were detected in 9 patients (9.5%). A 17 years old patient developed Takayasu arteritis after 12 years of follow-up.

#### Malignancy

Malignancy developed in 2 patients and both succumbed to it. A child diagnosed to have WAS at 8 months developed high grade intracranial non-Hodgkin lymphoma as early as 3.5 years (27) while the second child succumbed to non-Hodgkin lymphoma at 18 years of age. He had persistent monoclonal gammopathy without overt malignancy for 8 years prior to clinical evidence of the disease (28).

### Laboratory Investigations

#### Thrombocytopenia

Thrombocytopenia is a hallmark of WAS. Median platelet count in our cohort was 20 x10^9^/L (range 1 x10^9^/L to 142 x10^9^/L; IQR: 12 x10^9^/L – 32.2 x10^9^/L) was recorded in our cohort. Life threatening intracranial bleeding and gastrointestinal was recorded in 5 and 2 patients, respectively.

#### Mean Platelet Volume

MPV values were available for 65 patients and median MPV was 5.6 fL (range 3.1- 10.8 fL; IQR 5 - 7 Fl),. An MPV value of >7.5 fL was seen in 10/65 patients (15.3%) which could be attributed to differences in laboratory methods and techniques.

#### Serum Immunoglobulins

Details of estimated serum immunoglobulins (Ig) levels were available in 45 patients. Variable immunoglobulin profile was observed in these patients. Serum IgG was normal in 28 (62,2%), increased in 15 (33.4%) patients and was reduced in 2 (4.4%) patients. Low serum IgM was noted in 13 (28.8%) patients. Serum Ig A and Ig E were elevated 23 (51.1%) and 39 (87%) patients, respectively.

#### Lymphocyte Subset Analysis

Flow cytometric data of lymphocyte subset (CD3+ T cells, CD19+ B cells, CD56+ NK cells and CD3+CD56+ NKT cells) was available for 28 patients (**Supplementary Table 1**). Absolute lymphocyte counts (ALC) were low in 5/23 (%) patients, who had concomitant CMV infection or autoimmune hemolytic anemia or hemophagocytic lymphohistiocytosis (HLH), Median CD3+ T lymphocyte counts were 2.017 x10^9^/L (67.3%) (IQR 1.48- 3.59 x10^9^/L, range: 0.43 - 10.6 x10^9^/L), while median CD19+ B lymphocyte counts were 0.38x10^9^/L (10.44%) (IQR 0.154 - 0.716 x10^9^/L, range: 0.04 - 2.17 x10^9^/L). Median CD56+ NK lymphocyte counts were 0.436 x10^9^/L (12.24%) (IQR 0.176- 1 x10^9^/L, range: 0.1- 1.46 x10^9^/L. B lymphocytes were reduced in 11/20 (55%) patients while 8 patients had elevated levels of NK cells (36). Detailed immunophenotyping of subsets and T cell proliferation assay were not routinely carried out at most centers due to nonavailability.

#### WASp Expression by Flow Cytometry

Results of WASp expression on lymphocytes were available in 34 of 95 patients (6 patients with XLT and 28 patients with WAS). Reduced WASp expression (SI 0.29, 0.00 - 0.64) was seen in 30/34 patients (88.2%). Of these, 24 patients had WAS and 6 patients had XLT phenotype. Normal WASp (SI 0.75, 0.74 - 0.83) expression was observed in remaining 4 children. Of these 4 patients with normal WASp, 2 had intronic splice site mutation, and the remaining 2 patients had frameshift mutation in exon 10 (C-terminal end) of *WAS* gene.

#### Genetic Analysis

**Figure 2:**
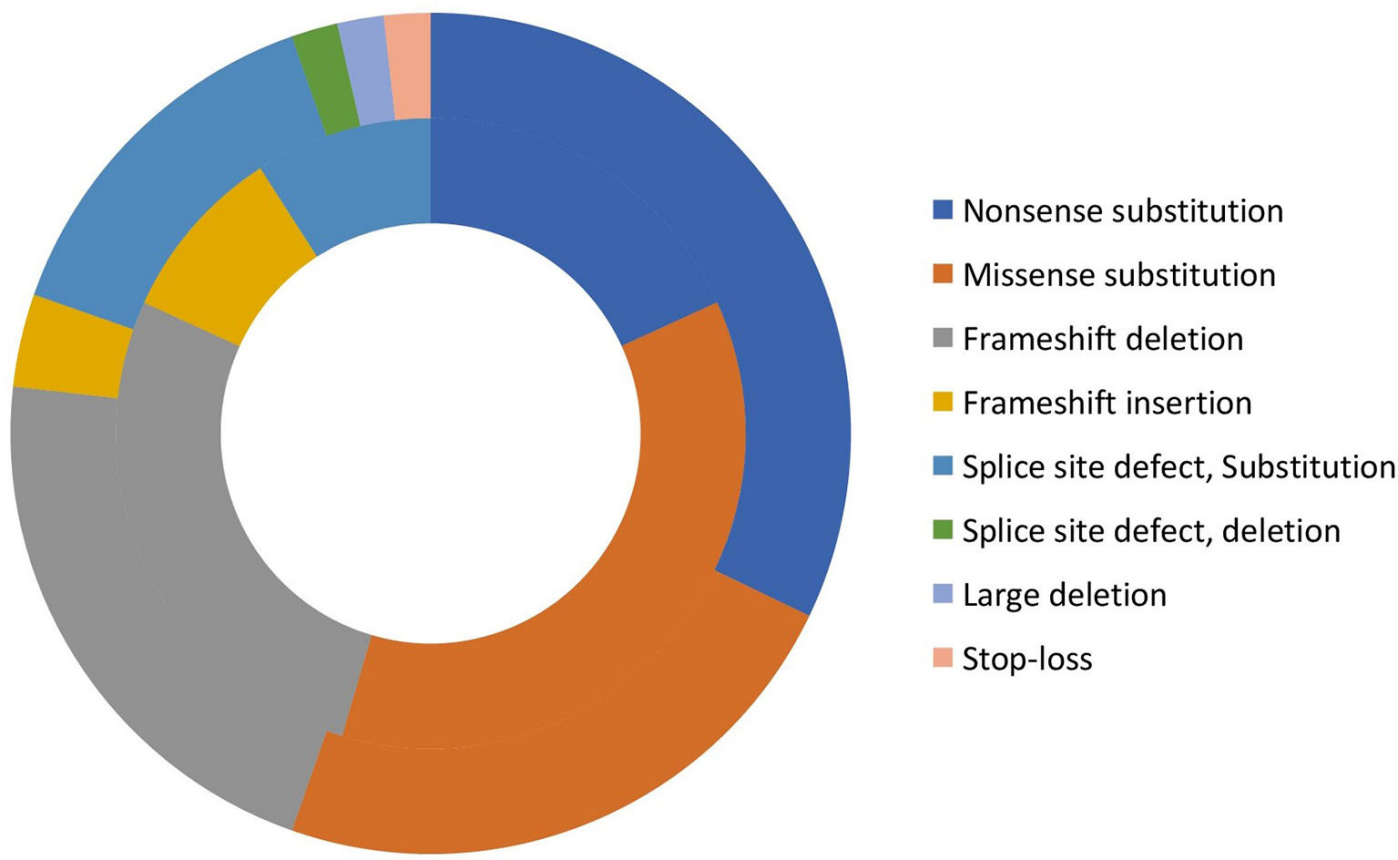
Distribution of variants in XLT, and classic WAS patients (Inner circle: XLT, and outer circle: WAS).

Eighty-six patients underwent DNA analysis; however, complete variant details were available for 67 patients at time of analysis of data. We identified 47 *WAS* gene variants in these 67 patients, 24 of which were novel and are being reported here for the first time (**Supplementary Table 2**). Mutations were found in all exons, except exons 8 and 11. Commonest mutations were single nucleotide substitutions (39/67) [nonsense (19 patients, 28.4%); missense (19 patients, 28.4%); and stop-loss (1 patient, 1.5%)]; followed by frameshift deletions (n=13, 19.4%); splice-site defects (n=11, 16.4%); small insertions (n=3, 4.5%); and large deletions (n=1, 1.5%). (**Supplementary Table 3** and **Figure 2**). Fifteen of 19 missense mutations were found in EVH1 (Drosophila enabled/vasodilator-stimulated phosphoprotein homology 1) domain (exons 1- 4). Hotspot mutations identified in our cohort included (p.R86H (n=5), p.R13X (n=4), p.R34X (n=4). It is interesting to note that, of the 6 frameshift deletions in exon 10, 5 resulted in termination of protein at 444 position (Patient no 03, 32,84,96,98,25,26 in **Supplementary Table 2**).

#### Novel Variants

**Figure 3:**
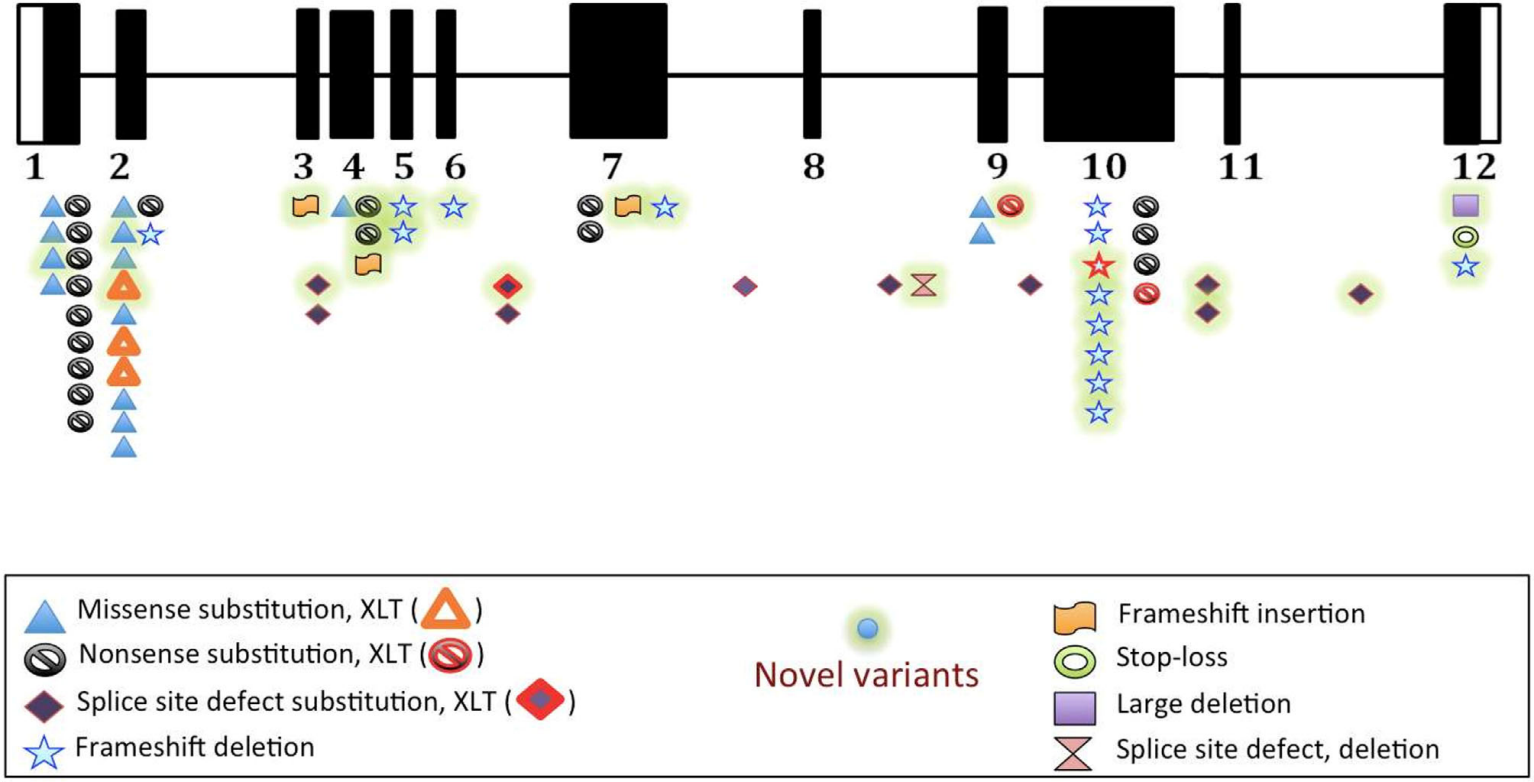
Distribution of variants on various exons, and intervening sequences of *WAS* gene in study population (n=67).

Twenty-four novel variants (marked with bold in **Supplementary Table 2** and **Figure 3**) were found in 27 patients. These included deletions (10); splice-site (5); insertions (3); nonsense (3); and missense (3). Four novel variants were found in proline rich region (exon 10) while 3 were in EVH1 domain (exon 4). Of the remaining 17 variants, 12 were exonic while 5 were intronic. Combined Annotation Dependent Depletion (CADD) Scores for single nucleotide variants (3 missense and 4 splice site) were calculated and was found to be more than 24 suggesting deleterious effect (**Supplementary Table 2**). Sixteen novel variants (frameshift deletions, frameshift insertions and nonsense) resulted in premature termination (PT) protein synthesis and were thus classified as pathogenic.

Missense and splice-site variants (7/9) occurred in the coiled-coil region of WASp protein. Missense variants (p.G40R, p.T48N and p.V75L) and splice-site variant (c.415+5G>C) affected WH1 domain responsible for binding of WASp interacting protein (WIP) and stabilization of WASp. Variant (p.G40R) resulted in substitution of non-polar amino acid by a polar amino acid at 40^th^ position placed adjacent to α-helix and resulted in reduction of Wasp protein. Stability of β-strands was affected by variants (p.T48N and p.V75L) as substituted amino acids were neither β-branched nor aromatic. Variant (p.T48N) also resulted in low WASp expression. Intervening sequence (IVS) variant (c.832+3_ +6delGAGT) caused splicing defect in Cdc42-binding site and Rac- interactive binding (CRIB) domain and reduced expression of WASp in expression.

Deletion (c.1564_1567delAGTG) affected Verprolin homology sequence (V) that binds monomeric actin and central (C) and acidic (A) sequence (VCA) domain caused extension of WASp beyond termination codon, binding of Actin related protein 2/3 (Arp2/3) complex resulting in instability of WASp. Variant (c.1507+2T>A) caused splicing defect and affected central (C) sequence essential for nucleation and interaction with Arp2/3 complex disturbing the stability of WASp and also affected WASp protein expression in the patient.

### Genotype-Phenotype Correlations

Of 67 patients in whom genetic analysis details were available, 56 had WAS and 11 had XLT (**Supplementary Table 3**). Nonsense variants (32.1%) were the most common mutations found in patients with WAS while missense mutations were most common mutations found in patients with XLT (4/11, 36.4%). Premature termination (non-sense, frameshift, and splice site mutation) was more frequent in patients with WAS phenotype than those with XLT [39/56 (69.6%) versus 7/11 (63.6%)]. Though, missense mutation were more common in XLT, 15 patients (26.7%) with WAS were found to have missense mutations.

### Treatment

Treatment details were available for 66 of 95 patients in our cohort. Of these 57 patients (86.3%) were commenced on cotrimoxazole prophylaxis. IVIg was administered in prophylactic dose (400 mg/kg/month) in 52 patients (78.8%). Twenty-eight patients (28/66, 42.4%) had received corticosteroids. Major indications of corticosteroid treatment included autoimmune manifestations like AIHA (8 patients), skin vasculitis (8 patients), and colitis (2 patients). Eight patients had received corticosteroids in variable doses and duration for refractory thrombocytopenia as they were initially diagnosed and followed up as ITP. Rituximab was used in the patient with Takayasu arteritis (and WAS) in addition to IVIg and methyl prednisolone. Splenectomy was performed only in one patient with refractory thrombocytopenia.

HSCT was performed in 25 patients (26.3%) **(** **Supplementary Table 4** **).** Haploidentical transplants were carried out in 10 patients while matched unrelated transplant were performed in 4 patients. Five patients each underwent matched related, and cord blood donor transplantation (**Supplementary Table 4** **)**. Conditioning regimens included fludarabine (n= 19), busulfan (n=14), and anti-thymocyte globulin (n = 10). Acute graft versus host disease (GVHD) occurred in 10 of 24 transplant recipients and 9 of these were managed successfully with systemic corticosteroids. One child succumbed to acute GVHD. Complete donor chimerism (whole blood donor cell chimerism of >95%) was attained in 15/25 recipients (60%), while mixed chimerism occurred in 3 patients. Successful lymphoid and myeloid engraftment was achieved in 14 patients, and they were maintaining a disease-free survival course (DFS) till date. One child was awaiting a second HSCT. Nine patients succumbed to transplant related complications- 4 had sepsis; 2 had GVHD; 1 each had CMV disease, pulmonary hemorrhage, acute respiratory distress syndrome, and immune cytopenia.

### Outcomes

Outcome details were available for 89 of 95 patients (93.7%), while follow-up duration was available for 56 patients with a median follow-up was 36 months (range 2 weeks- 12 years; IQR 16.2 months- 70 months). Of 89 patients in whom follow up data were available, 33 (37%) had died (classic WAS 32; XLT 1) at the time of analysis. Causes of death included intracranial hemorrhage (5 patients), transplant related complications (9 patients, **Supplementary Table 4**), malignancy (2 patients), severe gastrointestinal bleed (1 patient), HLH(1 patient), infections (7 patients including 2 patients with pneumonia). Cause of death could not be ascertained in remaining 8 patients.

### Survival Analysis

**Figure 4:**
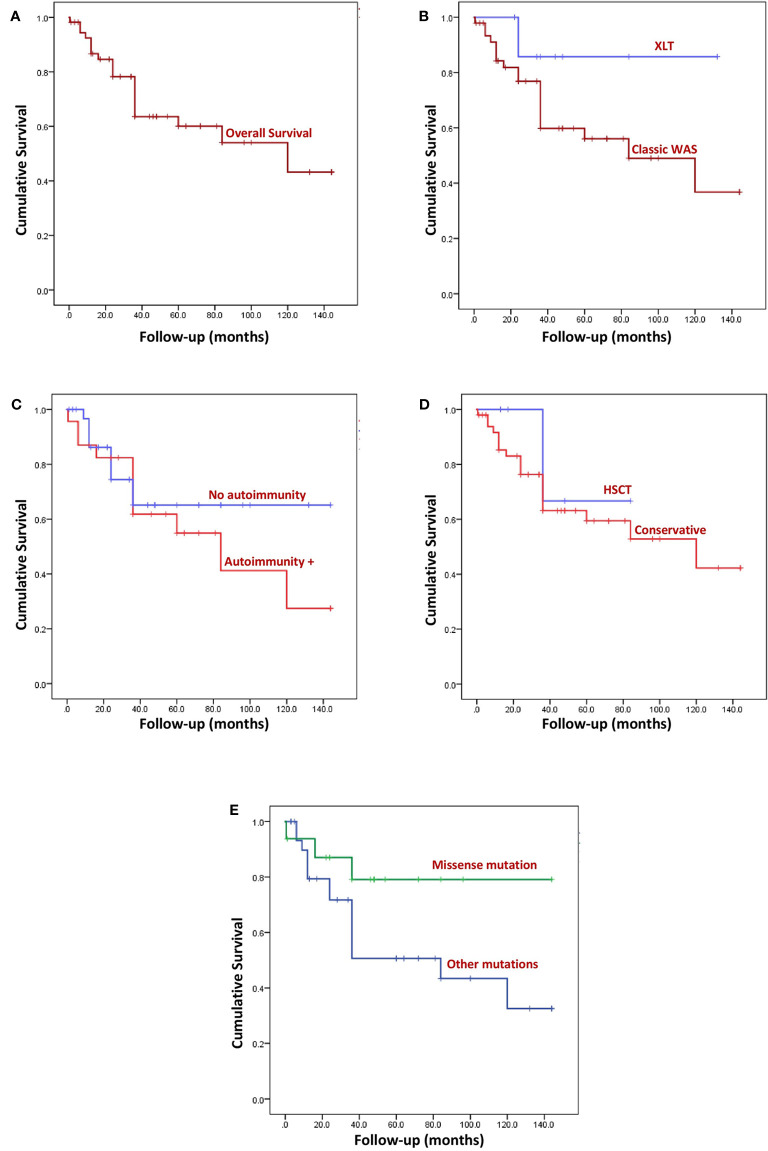
Kaplan Meier survival curves. **(A)** Overall survival (OS) of the entire cohort. Median follow-up was 36 months (range: 2 weeks- 12 years). **(B)** Comparison of OS between XLT and WAS, (p 0.154). **(C)** Comparison of OS of patients with autoimmune manifestation and without autoimmunity, (p 0.410). **(D)** Comparison of OS of patients who underwent HSCT and patients who were not transplanted, (p 0.543). **(E)** Comparison of OS of patients who had missense variants and those with variants other than missense mutation, (p 0.083).

Overall survival of our cohort is shown in **Figure 4A**. Cumulative survival on Kaplan- Meier analysis was not statistically significant between the following parameters: XLT versus classic WAS (P 0.154) (**Figure 4B**); presence or absence of autoimmune manifestations (p 0.410) (**Figure 4C**); HSCT versus conservative management (p 0.543) (**Figure 4D**); and missense mutations versus other types of mutations (p 0.083) (**Figure 4E**).

## Discussion

WAS is one of the oldest PIDs described, yet there are hardly any data on this condition from India. We report the first-ever multicentric cohort of children with WAS from India.

Clinical presentation of patients with WAS/XLT is extremely variable. Patients often present to different pediatric sub-specialties, and are often misdiagnosed as chronic ITP, colitis, or atopic dermatitis (37). It is therefore important that General pediatricians, as well as those working in pediatric sub-specialties should be aware of this disorder for early diagnosis. The classical triad of eczema, thrombocytopenia, and immune deficiency may not be evident at first presentation (38) but usually evolves over time. The triad was noted only in (15.7% patients in our cohort also at the time of first presentation.

The median age of diagnosis was as low as 1.75 months in an initial cohort from USA (38) while it ranged from 11months (1-42 months) to 24 months (1-132 months) in studies from China and Turkey respectively (37, 39). Mean age at diagnosis was 31.9 months (1-108 months) at our center in the year 2008 (23). However, median age at diagnosis in this multicentric cohort was found to be 12 months (5.5-36 months). This suggests improved recognition and early diagnosis of patients with WAS which can be ascribed to increased sensitization and improving diagnostic facilities in our country.

Thrombocytopenia is a universal feature of WAS and XLT, usually manifesting as petechiae, spontaneous or prolonged bleeding in first year of life. Most common presenting manifestations observed in our study was bleeding from gastrointestinal tract and skin bleeds as reported previously in other studies also (37, 38, 40). As us uniform graded data about bleeding was not available from all centers, A comparative profile of clinical manifestations with various published cohorts have been summarized in **Supplementary Table 5**.

Patients with WAS are more susceptible to infections due to impairment in both cellular and humoral immunity. Sino-pulmonary infections were the commonest infection in our cohort. Viral infections from Varicella-zoster virus (VZV), Herpes simplex virus (HSV), EBV, CMV, and Human papillomavirus (HPV) can be extremely severe CMV infection in WAS needs particular attention and often poses a diagnostic dilemma (38, 41–43). Thrombocytopenia may be erroneously ascribed to CMV infection and underlying diagnosis of WAS is often missed or delayed (41, 42). Life threatening hemorrhagic varicella was observed in 2 patients. Complicated varicella infection is often seen in children in India, as vaccination is optional and not part of universal immunization program. It is essential to highlight that all patients with WAS had received BCG vaccine at birth (as part of the national immunization schedule); however, BCG adenitis or disseminated BCG disease was not seen in any patient. BCG infection in patients with WAS per se is extremely rare (44).

Autoimmune manifestations are seen in up to 70% of WAS patients (37–40, 45–48) and can even occur post HSCT (49). A multitude of autoimmune complications affecting all organs (**Supplementary Table 5**) are described though autoimmune cytopenia (including hemolytic anemia, neutropenia, and thrombocytopenia) are the most reported complications. Autoimmune manifestations were seen in 40% of children, with AIHA being the commonest. Unusual autoimmune complication seen in our cohort were Takayasu arteritis (50) and Guillain Barre syndrome, which have been rarely described before (50).

Children with WAS have increased risk to develop malignancies. Overall incidence of tumors is reported in up to 13% (38, 46) while in our cohort it was 2.1%. Most reported malignancies are EBV related B cell lymphomas or leukemia (2, 38). As most children with WAS are transplanted early in developed countries malignancies are seen much less commonly. However, they are still encountered in patients in developing countries and are important contributors to mortality. Both the children with malignancy in our cohort succumbed to their illness.

Differentiation between XLT and WAS is based on the clinical scoring system (35). Clinical observations indicate that age of patient at diagnosis may influence the score assignment. Bleeding may be the only clinical manifestation in early infancy, suggesting a milder phenotype. These patients may evolve to develop serious infections, autoimmune manifestations, and malignancy later in childhood. Patients with milder phenotypes of WAS may go on to develop serious infections, autoimmune manifestations, and malignancy later in childhood. Also, clinical observations indicate that age of patient at diagnosis may influence the score assignment. Thus, it has been proposed to assign a score of 5 to patients with age below 2 years who present with life-threatening severe refractory thrombocytopenia (51). Five patients who were labeled as XLT evolved into the WAS phenotype on follow-up. Moreover, WAS expression or genotype is not considered in the scoring system.

Intracellular staining of WASp by flow cytometry is a quick test to establish diagnosis if protein expression is completely absent or markedly reduced. However, interpretation of result is difficult in patients with residual expression. Genetic analysis is needed in clinically suspected patients with residual WASp expression for confirmation of diagnosis. Despite expanding availability of flow cytometry-based test, this specialized test is not easily available at all centers in India. WASp expression may be preserved in patients with milder phenotypes with missense variants (46) and mutations involving C terminal end of *WAS* gene. WASp antibody usually recognizes and binds to epitope in the region between amino acid 146-265 thus resulting in normal WASp expression in some patients (52). Normal WASp levels could also be seen in patients with revertant mutations. Two patients with intronic mutations and two patients with frameshift mutation in C terminal (exon 10) had normal WASp expression in our cohort.

Identification of mutation in the *WAS* gene is essential for confirmation of diagnosis of WAS. Sanger sequencing technique is an easy, accessible and effective method for determining variants in the 12 exons and intron/exon boundaries of the WAS gene. Coverage of proximal and distal promoter regions of WAS gene is important as variants are often encountered in these regions and can be missed by NGS panels. We found 10 unique intronic mutations in 11 families in our cohort.

We found 47 unique mutations of *WAS* gene in 67 patients in our cohort. Of these, 24 proved to be novel. Fifty-six patients had exonic variants, with 31 patients (55.3%) having mutations localized to exons 1-4. Several cohorts have reported a frequency of 50%- 58.8% of these mutations in first 4 exons (40, 42–44, 51, 53, 54). This information is helpful for initial screening of patients by targeted sequencing. Studies suggested nonsense variants to be the commonest in their cohort (40, 42–44, 48, 51, 53–55), while others have found missense mutations to be more common (47, 56, 57). In our study both missense, and nonsense variants were equal.

Published literature suggests that deleterious mutations (i.e., non-sense, frameshift, and splice site mutations) are more common in classic WAS as compared to XLT phenotype (45). In our cohort, these mutations were 5.5 times more frequent in patients with classic WAS than XLT. However, this difference was not statistically significant (p 0.262). On the other hand, missense mutations which are reported more with XLT (45) were also found in 26.78% patients with WAS.

Management of patients with WAS must be individualized to specific clinical manifestations and degree of severity. Allogeneic HSCT has proven to be potentially curative and more recently, gene therapy is an important alternative in patients in whom HSCT cannot be performed (58). Conventionally, children with XLT, who have milder disease, do not require standard prophylactic interventions. However, with declining immune functions, development of clinically evident infections or defective antibody responses they too may require prophylactic measures such as cotrimoxazole prophylaxis or IVIg replacement. Risk of life -threatening complications and recognized life-long medical problems that affect the prognosis and quality of life of these patients, many centers now also support early HSCT for patients with XLT (59). However, risk-benefit of HSCT for patients with WAS mutations needs to be individualized in resource limited settings with high transplant related complications and mortality.

Decision to initiate chemoprophylaxis with cotrimoxazole (and whether it should include anti-viral and/or antifungal medications) depends on frequency, severity, and type of infections suffered by the individual patients. Most of our patients were initiated on cotrimoxazole prophylaxis (57 patients, 86.3%).

Prophylactic immune globulin treatment IVIg needs to be given even if serum IgG levels are within normal limits, as these patients have functional defects. Most of our patients at PGIMER, were initiated on replacement therapy depending upon frequency and severity of infections. Subcutaneous immunoglobulin is not available in India at present. Providing regular IVIg is a daunting task in financially deprived situations. Of late, support from state Governments and well as non-governmental philanthropic organizations has been very helpful is recognized in providing this therapy to children with PID.

WAS was among the first few diseases to be treated by HSCT (1968) (60). HSCT results in complete reversal of the disease and reported outcomes from developed countries are extremely encouraging. The ESID Registry of 170 patients with WAS have reported an overall survival of 87% with HLA-matched sibling donors (MSD), 71% with MUD, and 52% with mismatched donors (61). There are occasional reports of successful HSCT in patients with WAS from various centers in India (25, 26, 29). Only 25 patients (26.3%) could undergo HSCT in our cohort, and transplant related mortality was 36%. HSCT still remains out of reach for most patients in developing countries. There are many barriers to successful HSCT in resource limited settings. Limited centers offer this specialized therapy and there is a need for more robust donor registries. Delays in diagnosis often results in transplant being performed much later in life-this itself affects the outcomes adversely.

Mortality (33 patients, 37%) continues to be high in our cohort. Like threatening bleeding episodes, infections, and development of complications like malignancy and autoimmunity remain important causes of death.

Several limitations of the study are recognized. Complete uniform data was not available from all centers especially with respect to bleeding, infections, and treatment. Laboratory data including immunophenotyping of lymphocyte subsets, WASp expression and genetic analysis could not be performed in all patients. Moreover, the median follow-up was only 3 years only, thereby limiting the data about long term complications and, and outcomes.

The authors are aware of other centers in the country that could not participate in the study and have not been thus reported.

## Conclusions

To conclude, we document the first nationwide study on clinical and genetic features of 95 patients with WAS from India. We report wide spectrum of clinical manifestations and 24 novel variants in our cohort. Mortality continues to be high as definitive therapy is not accessible to all patients. There is an urgent need to increase awareness of WAS amongst internists and pediatricians and improve pediatric HSCT services in our country.

## Data Availability Statement

The original contributions presented in the study are included in the article/**Supplementary Material**, further inquiries can be directed to the corresponding author.

## Ethics Statement

The studies involving human participants were reviewed and approved by Institute Ethics Committee, PGIMER, Chandigarh. Written informed consent from the participants' legal guardian/next of kin was not required to participate in this study in accordance with the national legislation and the institutional requirements. Written informed consent was obtained from the minor(s)' legal guardian/next of kin for the publication of any potentially identifiable images or data included in this article.

## Author Contributions

DS, RRi, AJ, PV, AG, MS, AKh, RRa, RU, MD, PT, VG, AP, MM, HL, SB, HK, SV, and SS: Clinical management of patients and provided necessary clinical details for compilation. AR, MM, JA, RR, PB, AKa, JS, PT, KI, KC, PL, OO, SN, and YL: Laboratory work-up of patients and provided necessary laboratory results for compilation. DS, RRi, MS, AKa, and JS: compiled the data and framed the initial draft and editing of manuscript. DS, RRi, MS, and AKa: literature search. DS and SS: editing of manuscript at all stages of preparation and final approval. All authors contributed to the article and approved the submitted version.

## Funding

This work was supported by India Council of Medical Research (ICMR), New Delhi, India, and Department of Health Research, Ministry of Health and Family Welfare, Government of India, New Delhi, India for funding (vide Grant No. GIA/48/2014-DHR). 2.Department of Biotechnology, Ministry of Science and Technology, Government of India (DBT/PR26412/MED/12/792/2017). However, the funders had no role in study design, data collection and analysis, decision to publish, or preparation of the manuscript.

## Conflict of Interest

The authors declare that the research was conducted in the absence of any commercial or financial relationships that could be construed as a potential conflict of interest.

## References

[1] Ryser O Morell A Hitzig WH. Primary immunodeficiencies in Switzerland: first report of the national registry in adults and children. J Clin Immunol (1988) 8(6):479–85. 10.1007/BF00916954 3065352

[2] Perry GS3 Spector BD Schuman LM Mandel JS Anderson VE McHugh RB. The Wiskott-Aldrich syndrome in the United States and Canada (1892-1979). J Pediatr (1980) 97:72–8. 10.1016/s0022-3476(80)80133-8 7381651

[3] Ochs HD Thrasher AJ. The Wiskott-Aldrich syndrome. J Allergy Clin Immunol (2006) 117:725–38. 10.1016/j.jaci.2006.02.005 quiz 739. 16630926

[4] Ochs HD Filipovich AH Veys P Cowan MJ Kapoor N. Wiskott-Aldrich syndrome: diagnosis, clinical and laboratory manifestations, and treatment. Biol Blood Marrow Transplant (2009) 15:84–90. 10.1016/j.bbmt.2008.10.007 19147084

[5] Kwan SP Lehner T Hagemann T Lu B Blaese M Ochs H. Localization of the gene for the Wiskott-Aldrich syndrome between two flanking markers, TIMP and DXS255, on Xp11.22-Xp11.3. Genomics (1991) 10:29–33. 10.1016/0888-7543(91)90480-3 1675197

[6] Rivero-Lezcano OM Marcilla A Sameshima JH Robbins KC. Wiskott-Aldrich syndrome protein physically associates with Nck through Src homology 3 domains. Mol Cell Biol (1995) 15:5725–31. 10.1128/mcb.15.10.5725 PMC230823 7565724

[7] Symons M Derry JM Karlak B Jiang S Lemahieu V Mccormick F. Wiskott-Aldrich syndrome protein, a novel effector for the GTPase CDC42Hs, is implicated in actin polymerization. Cell (1996) 84:723–34. 10.1016/s0092-8674(00)81050-8 8625410

[8] Miki H Nonoyama S Zhu Q Aruffo A Ochs HD Takenawa T. Tyrosine kinase signaling regulates Wiskott-Aldrich syndrome protein function, which is essential for megakaryocyte differentiation. Cell Growth Differ (1997) 8:195–202. 9040941

[9] Miki H Takenawa T. Direct binding of the verprolin-homology domain in N-WASP to actin is essential for cytoskeletal reorganization. Biochem Biophys Res Commun (1998) 243:73–8. 10.1006/bbrc.1997.8064 9473482

[10] Parolini O Berardelli S Riedl E Bello-Fernandez C Strobl H Majdic O. Expression of Wiskott-Aldrich syndrome protein (WASP) gene during hematopoietic differentiation. Blood (1997) 1; 90:70–5. 10.1182/blood.V90.1.70.70_70_75 9207440

[11] Cory GO MacCarthy-Morrogh L Banin S Gout I Brickell PM Levinsky RJ. Evidence that the Wiskott-Aldrich syndrome protein may be involved in lymphoid cell signaling pathways. J Immunol (1996) 157:3791–5. 8892607

[12] Gallego MD Santamaría M Peña J Molina IJ. Defective actin reorganization and polymerization of Wiskott-Aldrich T cells in response to CD3-mediated stimulation. Blood (1997) 90:3089–97. 10.1182/blood.V90.8.3089 9376590

[13] Krawczyk C Bachmaier K Sasaki T Jones RG Snapper SB Bouchard D. Cbl-b is a negative regulator of receptor clustering and raft aggregation in T cells. Immunity (2000) 13:463–73. 10.1016/s1074-7613(00)00046-7 11070165

[14] Sasahara Y Rachid R Byrne MJ de la Fuente MA Abraham RT Ramesh N. Mechanism of recruitment of WASP to the immunological synapse and of its activation following TCR ligation. Mol Cell (2002) 10:1269–81. 10.1016/s1097-2765(02)00728-1 12504004

[15] Dupré L Aiuti A Trifari S Martino S Saracco P Bordignon C. Wiskott-Aldrich syndrome protein regulates lipid raft dynamics during immunological synapse formation. Immunity (2002) 17:157–66. 10.1016/s1074-7613(02)00360-6 12196287

[16] Orange JS Ramesh N Remold-O’Donnell E Sasahara Y Koopman L Byrne M. Wiskott-Aldrich syndrome protein is required for NK cell cytotoxicity and colocalizes with actin to NK cell-activating immunologic synapses. Proc Natl Acad Sci U.S.A. (2002) 99:11351–6. 10.1073/pnas.162376099 PMC123260 12177428

[17] Nikolov NP Shimizu M Cleland S Bailey D Aoki J Strom T. Systemic autoimmunity and defective Fas ligand secretion in the absence of the Wiskott-Aldrich syndrome protein. Blood (2010) 116:740–7. 10.1182/blood-2009-08-237560 PMC2918330 20457871

[18] Zhang J Shehabeldin A da Cruz LA Butler J Somani AK McGavin M. Antigen receptor-induced activation and cytoskeletal rearrangement are impaired in Wiskott-Aldrich syndrome protein-deficient lymphocytes. J Exp Med (1999) 190:1329–42. 10.1084/jem.190.9.1329 PMC2195687 10544204

[19] Lorenzi R Brickell PM Katz DR Kinnon C Thrasher AJ. Wiskott-Aldrich syndrome protein is necessary for efficient IgG-mediated phagocytosis. Blood (2000) May 1 95(9):2943–6. 10.1182/blood.V95.9.2943.009k17_2943_2946 10779443

[20] Linder S Higgs H Hüfner K Schwarz K Pannicke U Aepfelbacher M. The polarization defect of Wiskott-Aldrich syndrome macrophages is linked to dislocalization of the Arp2/3 complex. J Immunol (2000) 165:221–5. 10.4049/jimmunol.165.1.221 10861055

[21] Jones GE Zicha D Dunn GA Blundell M Thrasher A. Restoration of podosomes and chemotaxis in Wiskott-Aldrich syndrome macrophages following induced expression of WASp. Int J Biochem Cell Biol (2002) 34:806–15. 10.1016/s1357-2725(01)00162-5 11950596

[22] Badolato R Sozzani S Malacarne F Bresciani S Fiorini M Borsatti A. Monocytes from Wiskott-Aldrich patients display reduced chemotaxis and lack of cell polarization in response to monocyte chemoattractant protein-1 and formyl-methionyl-leucyl-phenylalanine. J Immunol (1998) 161:1026–33. 9670984

[23] Suri D Singh S Rawat A Gupta A Kamae C Honma K. Clinical profile and genetic basis of Wiskott-Aldrich syndrome at Chandigarh, North India. Asian Pac J Allergy Immunol (2012) 30:71–8. 22523910

[24] David S Jayandharan GR Abraham A Jacob RR Devi GS Patkar N. Molecular basis of Wiskott-Aldrich syndrome in patients from India. Eur J Haematol (2012) 89:356–60. 10.1111/j.1600-0609.2012.01818.x.Epub2012Jul14 22679904

[25] Uppuluri R Jayaraman D Sivasankaran M Patel S Swaminathan VV Vaidhyanathan L. Hematopoietic Stem Cell Transplantation for Primary Immunodeficiency Disorders: Experience from a Referral Center in India. Indian Pediatr (2018) 55:661–4. 10.1007/s13312-018-1354-9 30218511

[26] Uppuluri R Sivasankaran M Patel S Swaminathan VV Ramanan KM Ravichandran N. Haploidentical Stem Cell Transplantation with Post-Transplant Cyclophosphamide for Primary Immune Deficiency Disorders in Children: Challenges and Outcome from a Tertiary Care Center in South India. J Clin Immunol (2019) 39:182–7. 10.1007/s10875-019-00600-z PMC7100782 30778805

[27] Vignesh P Suri D Rawat A Lau YL Bhatia A Das A. Sclerosing cholangitis and intracranial lymphoma in a child with classical Wiskott-Aldrich syndrome. Pediatr Blood Cancer (2017) 64:106–9. 10.1002/pbc.26196 27566838

[28] Rikhi R Bhattad S Jindal A Saikia B Garg R Rawat A. Monoclonal Gammopathy of Unclear Significance in a Child with Wiskott-Aldrich Syndrome: a Rare Occurrence. J Clin Immunol (2019) 39:7–10. 10.1007/s10875-018-0585-9 30607662

[29] John MJ Philip CC Mathew A Williams A Kakkar N. Un-manipulated Haploidentical Transplant in Wiskott-Aldrich Syndrome. Indian Pediatr (2017) 54(4):327–8. 10.1007/s13312-017-1097-z 28474594

[30] Gupta MC Agarwal VK Mittal AK Rajvanshi VS. Wiskott-Aldrich Syndrome. A Case Rep J Assoc Physicians India (1964) 12:531–3. 14184097

[31] Mathew LG Chandy M Dennison D Srivastava A Ganapathy K Cherian T. Successful bone marrow transplantation in an infant with Wiskott-Aldrich syndrome. Indian Pediatr (1999) 36(7):707–10. 10740310

[32] Srivastava A Swaid HA Kabra M Verma IC. Management of Wiskott-Aldrich syndrome. Indian J Pediatr (1996) 63(5):709–12. 10.1007/BF02730830 10830048

[33] Jindal AK Pilania RK Rawat A Singh S. Primary Immunodeficiency Disorders in India-A Situational Review. Front Immunol (2017) 8:714. 10.3389/fimmu.2017.00714 28674536 PMC5474457

[34] John MJ Philip CC Mathew A Williams A Kakkar N. Un-manipulated Haploidentical Transplant in Wiskott-Aldrich Syndrome. Indian Pediatr (2017) 54(4):327–328. 10.1007/s13312-017-1097-z 28474594

[35] Zhu Q Watanabe C Liu T Hollenbaugh D Blaese RM Kanner SB. Wiskott-Aldrich syndrome/X-linked thrombocytopenia: WASP gene mutations, protein expression, and phenotype. Blood (1997) 90:2680–9. 10.1182/blood.V90.7.2680.2680_2680_2689 9326235

[36] Shearer WT Rosenblatt HM Gelman RS Oyomopito R Plaeger S Stiehm ER. Pediatric AIDS Clinical Trials GroupLymphocyte subsets in healthy children from birth through 18 years of age: the Pediatric AIDS Clinical Trials Group P1009 study. J Allergy Clin Immunol (2003) 112(5):973–80. 10.1016/j.jaci.2003.07.003 14610491

[37] Haskoloğlu Ş Öztürk A Öztürk G Kostel Bal S İslamoğlu C Baskın K. Clinical Features and Outcomes of 23 Patients with Wiskott-Aldrich Syndrome: A Single-Center Experience. Turk J Haematol (2020) Nov 19 37(4):271–81. 10.4274/tjh.galenos.2020.2020.0334 PMC7702658 32812413

[38] Sullivan KE Mullen CA Blaese RM Winkelstein JA. A multiinstitutional survey of the Wiskott-Aldrich syndrome. J Pediatr (1994) 125:876–85. 10.1016/s0022-3476(05)82002-5 7996359

[39] Lee PP Chen TX Jiang LP Chen J Chan KW Lee TL. Clinical and molecular characteristics of 35 Chinese children with Wiskott-Aldrich syndrome. J Clin Immunol (2009) 29(4):490–500. 10.1007/s10875-009-9285-9 19308710

[40] Jin YY Wu J Chen TX Chen J. When *WAS* Gene Diagnosis Is Needed: Seeking Clues Through Comparison Between Patients with Wiskott-Aldrich Syndrome and Idiopathic Thrombocytopenic Purpura? Front Immunol (2019) 10: 015 49: 015 49. 10.3389/fimmu.2019.01549 PMC6634258 31354712

[41] Kaneko R Yamamoto S Okamoto N Akiyama K Matsuno R Toyama D. Wiskott-Aldrich syndrome that was initially diagnosed as immune thrombocytopenic purpura secondary to a cytomegalovirus infection. SAGE Open Med Case Rep (2018) 6:2050313X17753788. 10.1177/2050313X17753788 PMC5768273 29348920

[42] Poddighe D Virginia E Nedbal M Soresina A Bruni P. Postnatal cytomegalovirus infection in an infant with congenital thrombocytopenia: how it can support or mislead the diagnosis of Wiskott-Aldrich syndrome. Infez Med (2016) 24:237–40. 27668906

[43] Kaushik S Singh R Gupta A Singh S Suri D Sarpal N. Unilateral recalcitrant glaucoma associated with cytomegalovirus in an immunocompromised child with Wiskott-Aldrich syndrome. J AAPOS (2013) 17:646–7. 10.1016/j.jaapos.2013.08.007 24210345

[44] Reichenbach J Rosenzweig S Döffinger R Dupuis S Holland SM Casanova JL. Mycobacterial diseases in primary immunodeficiencies. Curr Opin Allergy Clin Immunol (2001) 1:503–11. 10.1097/00130832-200112000-00003 11964733

[45] Albert MH Bittner TC Nonoyama S Notarangelo LD Burns S Imai K. X-linked thrombocytopenia (XLT) due to WAS mutations: clinical characteristics, long-term outcome, and treatment options. Blood (2010) 115(16):3231–8. 10.1182/blood-2009-09-239087 20173115

[46] Imai K Morio T Zhu Y Jin Y Itoh S Kajiwara M. Clinical course of patients with WASP gene mutations. Blood (2004) 103(2):456–64. 10.1182/blood-2003-05-1480 12969986

[47] Shin CR Kim MO Li D Bleesing JJ Harris R Mehta P. Excellent outcomes following hematopoietic cell transplantation for Wiskott-Aldrich syndrome: a PIDTC report. Blood (2020) 135(23):2094–105. 10.1182/blood.2019002939 PMC7273831 32268350

[48] Dupuis-Girod S Medioni J Haddad E Quartier P Cavazzana-Calvo M Le Deist F. Autoimmunity in Wiskott-Aldrich syndrome: risk factors, clinical features, and outcome in a single-center cohort of 55 patients. Pediatrics (2003) 111:e622–7. 10.1542/peds.111.5.e622 12728121

[49] Ozsahin H Le Deist F Benkerrou M Cavazzana-Calvo M Gomez L Griscelli C. Bone marrow transplantation in 26 patients with Wiskott-Aldrich syndrome from a single center. J Pediatr (1996) 129(2):238–44. 10.1016/s0022-3476(96)70248-2 8765621

[50] Lau YL Wong SN Lawton WM. Takayasu’s arteritis associated with Wiskott-Aldrich syndrome. J Paediatr Child Health (1992) 28(5):407–9. 10.1111/j.1440-1754.1992.tb02703.x 1356386

[51] Mahlaoui N Pellier I Mignot C Jais JP Bilhou-Nabéra C Moshous D. Characteristics and outcome of early-onset, severe forms of Wiskott-Aldrich syndrome. Blood (2013) 121:1510–6. 10.1182/blood-2012-08-448118 23264593

[52] Chiang SCC Vergamini SM Husami A Neumeier L Quinn K Ellerhorst T. Screening for Wiskott-Aldrich syndrome by flow cytometry. J Allergy Clin Immunol (2018) 142(1):333–335.e8. 10.1016/j.jaci.2018.04.017 29729304

[53] Gulácsy V Freiberger T Shcherbina A Pac M Chernyshova L Avcin T. Genetic characteristics of eighty-seven patients with the Wiskott-Aldrich syndrome. Mol Immunol (2011) 48:788–92. 10.1016/j.molimm.2010.11.013 21185603

[54] Jin Y Mazza C Christie JR Giliani S Fiorini M Mella P. Mutations of the Wiskott-Aldrich Syndrome Protein (WASP): hotspots, effect on transcription, and translation and phenotype/genotype correlation. Blood (2004) 104(13):4010–9. 10.1182/blood-2003-05-1592 15284122

[55] Burroughs LM Petrovic A Brazauskas R Liu X Griffith LM Ochs HD. Excellent outcomes following hematopoietic cell transplantation for Wiskott-Aldrich syndrome: a PIDTC report. Blood (2020) 135(23):2094–105. 10.1182/blood.2019002939 PMC7273831 32268350

[56] Shekhovtsova Z Bonfim C Ruggeri A Nichele S Page K AlSeraihy A. A risk factor analysis of outcomes after unrelated cord blood transplantation for children with Wiskott-Aldrich syndrome. Haematologica (2017) 102:1112–9. 10.3324/haematol.2016.158808 PMC5451344 28255019

[57] Lee WI Huang JL Jaing TH Wu KH Chien YH Chang KW. Clinical aspects and genetic analysis of taiwanese patients with wiskott-Aldrich syndrome protein mutation: the first identification of x-linked thrombocytopenia in the chinese with novel mutations. J Clin Immunol (2010) 30:593–601. 10.1007/s10875-010-9381-x 20232122

[58] Ferrua F Cicalese MP Galimberti S Giannelli S Dionisio F Barzaghi F. Lentiviral haemopoietic stem/progenitor cell gene therapy for treatment of Wiskott-Aldrich syndrome: interim results of a non-randomised, open-label, phase 1/2 clinical study. Lancet Haematol (2019) 6:e239–53. 10.1016/S2352-3026(19)30021-3 PMC6494976 30981783

[59] Oshima K Imai K Albert MH Bittner TC Strauss G Filipovich AH. Hematopoietic Stem Cell Transplantation for X-Linked Thrombocytopenia with Mutations in the WAS gene. J Clin Immunol (2015) 35(1):15–21. 10.1007/s10875-014-0105-5 25388447

[60] Bach FH Albertini RJ Joo P Anderson JL Bortin MM. Bone-marrow transplantation in a patient with the Wiskott-Aldrich syndrome. Lancet (1968) 2:1364–6. 10.1016/s0140-6736(68)92672-x 4177931

[61] Filipovich AH Stone JV Tomany SC Ireland M Kollman C Pelz CJ. Impact of donor type on outcome of bone marrow transplantation for Wiskott-Aldrich syndrome: collaborative study of the International Bone Marrow Transplant Registry and the National Marrow Donor Program. Blood (2001) 97:1598–603. 10.1182/blood.v97.6.1598 11238097

