# Supplementary material for: Wiskott Aldrich Syndrome: A Multi-Institutional Experience From India
Source: Front Immunol. 2021 Apr 16;12:627651. doi: 10.3389/fimmu.2021.627651 (PMC8086834; doi:10.3389/fimmu.2021.627651)
Supplement: Supplementary file 1 [file DataSheet_1.docx]

Appendix 1:

PCR conditions:

Each amplification reaction will contain 70-120 ng of high molecular weight DNA, 1.5 mM MgCl_2_ (Fermentas, United States), 0.25-0.5 mM of dNTP mix (dATP, dCTP, dGTP, and dTTP) (Fermentas, United States), 10 pmol of each primer (Sigma, United States) and 1.25 unit of Pfu DNA polymerase (Fermentas, United States) in a final volume of 20 μl. The PCR cycle explained briefly, initial denaturation at **95 ^ο^ C** for 5 min**,** followed by 34 cycles of **95^ο^C** for 30 second, annealing at different temperature for 30 seconds and extension at **72^ο^ C** at different time intervals for different primers and final extension at **72^ο^ C** for 10 min**.** PCR amplicons will be analyzed on 1.5 to 2% gel electrophoresis with reference to DNA ladder (100 bp, Fermentas, United States). The primer sequences (taken from RAPID Database) (1) are listed in **Supplementary Table 1**
