# Supplementary material for: Wiskott Aldrich Syndrome: A Multi-Institutional Experience From India
Source: Front Immunol. 2021 Apr 16;12:627651. doi: 10.3389/fimmu.2021.627651 (PMC8086834; doi:10.3389/fimmu.2021.627651)
Supplement: Supplementary file 2 [file DataSheet_2.docx]

**Annexe 1**

Patient ID and Name

Age at onset of symptoms (months)

Age at diagnosis (months)

Delay in diagnosis (months)

Age at study

**Initial presenting manifestations**

Bleeding (site/ severe bleeding manifestations)

Infections (Organ system/ microorganisms)

Thrombocytopenia

Eczema

Autoimmune manifestations (Vasculitis/ autoimmune hemolytic anemia/ arthritis/ colitis)

Others including malignancy

Patients with all 3 features of triad at initial presentation

**Family history**

**Clinical manifestations over the course of disease**

Bleeding (No of sites/ severe bleeding manifestations)

Infections and the spectrum of organ involvement

Patients had all 3 features of triad over the follow-up period

Autoimmune manifestations

Malignancy

**WAS clinical severity score**

**Investigations**

Mean platelet volume

Immunoglobulin Profile

Serum IgE levels

WASp expression

Lymphocyte subset analysis

T cell proliferation assay

Molecular analysis

**Treatment received**

Intravenous Immunoglobulin

Cotrimoxazole prophylaxis

Corticosteroids

Thrombopoietin receptor agonists

Hematopoietic stem cell transplant (HSCT)

Type of transplant

Engraftment

Complications due to HSCT

**Outcome**

Alive at the time of analysis

Died at the time of analysis

Lost to follow-up

**Follow up (months)**
