# Supplementary material for: Wiskott Aldrich Syndrome: A Multi-Institutional Experience From India
Source: Front Immunol. 2021 Apr 16;12:627651. doi: 10.3389/fimmu.2021.627651 (PMC8086834; doi:10.3389/fimmu.2021.627651)
Supplement: Supplementary file 3 [file Table_1.docx]

| S. No | ALC (x10^9^/L) | CD3+ T cell  (%) | CD3+ T cell (x10^9^/L) | Normal/ Low | CD19+ B cell  (%) | CD19+ B cell (x10^9^/L) | Normal/ Low | CD56+ NK cell  (%) | CD56+ NK  cell (x10^9^/L) | Normal/ Low | CD3+56+ NKT cell  (%) | CD3+ CD56+ NKT cell (x10^9^/L) | Normal/ Low |
| --- | --- | --- | --- | --- | --- | --- | --- | --- | --- | --- | --- | --- | --- |
| 1 | 5.742 | 62.53 | 3.59 | N | 12.47 | 0.72 | N | n/a | - | - | n/a | - | - |
| 2 | n/a | 41.07 | - | N | 34.01 | - | N | 22.51 | - | N | n/a | - | - |
| 3 | 3.35 | 52.17 | 1.75 | N | 16.06 | 0.54 | N | 24.24 | 0.81 | H | 2.13 | 0.07 | N |
| 4 | n/a | 71.08 | - | N | 20.65 | - | N | n/a | - | - | n/a | n/a | - |
| 5 | 5.004 | 74.00 | 3.70 | N | 13.00 | 0.65 | N | n/a | - | - | n/a | n/a | - |
| 6 | 0.624 | 69.42 | 0.43 | L | 6.00 | 0.04 | L | 20.33 | 0.13 | N | 2.33 | 0.01 | N |
| 7 | 15.038 | 70.50 | 10.60 | H | 14.40 | 2.17 | N | 7.10 | 1.07 | H | - | - | - |
| 8 | 5.7 | 77.00 | 4.39 | N | 12.44 | 0.71 | N | 7.51 | 0.43 | N | 1.17 | 0.07 | N |
| 9 | 1.045 | 68.88 | 0.72 | L | 14.81 | 0.15 | L | 12.74 | 0.13 | N | 0.69 | 0.01 | N |
| 10 | 3.84 | 57.93 | 2.22 | N | 6.12 | 0.24 | L | 2.68 | 0.10 | N | 26.68 | 1.02 | H |
| 11 | 2.5 | 59.99 | 1.50 | N | 5.06 | 0.13 | L | 9.58 | 0.24 | N | 20.98 | 0.52 | H |
| 12 | 1.48 | 80.48 | 1.19 | L | 5.35 | 0.08 | L | 11.74 | 0.17 | N | 0.86 | 0.01 | N |
| 13 | 3.48 | 75.59 | 2.63 | N | 7.44 | 0.26 | N | 11.65 | 0.41 | N | 4.93 | 0.17 | N |
| 14 | 1.131 | 41.73 | 0.47 | L | 5.80 | 0.07 | L | 41.51 | 0.47 | N | 2.71 | 0.03 | N |
| 15 | 2.736 | 73.75 | 2.02 | N | 16.29 | 0.45 | N | 6.74 | 0.18 | N | 1.00 | 0.03 | N |
| 16 | 2.354 | 72.01 | 1.70 | N | 9.75 | 0.23 | L | 10.56 | 0.25 | N | 6.48 | 0.15 | N |
| 17 | 2.58 | 59.97 | 1.55 | N | 14.75 | 0.38 | N | 21.05 | 0.54 | H | 3.00 | 0.08 | N |
| 18 | 2.222 | 66.64 | 1.48 | N | 8.32 | 0.18 | L | 20.04 | 0.45 | N | 1.66 | 0.04 | N |
| 19 | n/a | 63.60 | - | N | 20.67 | - | N | 14.35 | - | - | 0.28 | - | N |
| 20 | 4.746 | 67.87 | 3.22 | N | 15.33 | 0.73 | N | 11.71 | 0.56 | N | 1.16 | 0.06 | N |
| 21 | 13.138 | 69.40 | 9.12 | N | 9.24 | 1.21 | N | 10.98 | 1.44 | H | 7.42 | 0.97 | H |
| 22 | 5.246 | 50.00 | 2.62 | N | 13.94 | 0.73 | N | 24.17 | 1.27 | H | 4.11 | 0.22 | N |
| 23 | n/a | 61.95 | - | - | 7.61 | - | N | 21.67 | - | H | 7.37 | - | - |
| 24 | 3.537 | 43.83 | 1.55 | N | 5.89 | 0.21 | L | 41.15 | 1.46 | H | 4.56 | 0.16 | N |
| 25 | 6.129 | 58.73 | 3.60 | N | 7.63 | 0.47 | N | 2.17 | 0.13 | N | 29.67 | 1.82 | H |
| 26 | 1.429 | 80.37 | 1.15 | N | 3.04 | 0.04 | L | - | - | - | - | - | - |
| 27 | 7.144 | 64.72 | 4.62 | N | 11.14 | 0.80 | N | 19.03 | 1.36 | H | 1.46 | 0.10 | N |
| 28 | n/a | 85.57 | - | - | 5.07 | - | L | 0.5.51 | - | - | 1.82 | - | - |

Supplementary Table 1: Details of lymphocyte subsets of patients with WAS.

Abbreviations: ALC: Absolute lymphocyte count; CD3+ cells: T lymphocytes; CD19+: B lymphocytes; CD56+: NK lymphocytes; CD3+CD56+: NKT cells; N: Normal range; L: Less than normal; H: Higher than the upper limit of normal range; n/a: not-available.
