# Supplementary material for: Wiskott Aldrich Syndrome: A Multi-Institutional Experience From India
Source: Front Immunol. 2021 Apr 16;12:627651. doi: 10.3389/fimmu.2021.627651 (PMC8086834; doi:10.3389/fimmu.2021.627651)
Supplement: Supplementary file 4 [file Table_2.docx]

Supplementary table 2: Details of the variants found in patients with Wiskott-Aldrich syndrome (n=67).

| **Sr No** | **Patient ID** | **Onset of symptoms (months)** | **Age at diagnosis (months)** | **WAS Score** | **Exon** | **cDNA position** | **Protein change** | **Type of change** | **PT or CADD Score of SNV for Novel variants** | **WASp protein expression** | **Outcome** |
| --- | --- | --- | --- | --- | --- | --- | --- | --- | --- | --- | --- |
|  | 87 | 3 | 24 | 3 | 1 | c.92C>T | p.R13X | Nonsense substitution |  | N.D | Well |
|  | 13 | 6 | 12 | 4 |  |  |  |  |  | N.D | Expired |
|  | 14 | 0.2 | 5 | 4 |  |  |  |  |  | N.D | Expired |
|  | 88 | 5 | 12 | 5 |  |  |  |  |  | N.D | Well |
|  | 27 | 6 | 7 | 3 | 1 | c.147G>A | p.E31K | Missense substitution |  | Reduced | Expired |
|  | 28 | 2 | 6 | 5 |  |  |  |  |  | Reduced | Well |
|  | 78 | 3 | 20 | 3 | 1 | c.155C>T | p.R34X | Nonsense substitution |  | N.D | Well |
|  | 6 | 1 | 4 | 4 |  |  |  |  |  | N.D | Expired |
|  | 38 | 1 | 2 | 4 |  |  |  |  |  | Reduced | Expired |
|  | 1 | 0.3 | 4.5 | 5 |  |  |  |  |  | N.D | Well |
|  | 34 | 36 | 60 | 5 | 1 | c.170T>C | p.L39P | Missense substitution |  | Reduced | Well |
|  | **37** | **24** | **48** | **3** | **1** | **c.173G>A** | **p.G40R** | **Missense substitution** | **36 (48683971; GRCh38-v1.6)** | **Reduced** | **Well** |
|  | 7 | 4 | 18 | 5 | 1 | c.176C>T | p.R41X | Nonsense substitution |  | Absent | Well |
|  | 11* | 60 | 108 | 2 | 2 | c.189C>T | p.T45M | Missense substitution |  | N.D | Well |
|  | 10* | 60 | 72 | 4 |  |  |  |  |  | N.D | Well |
|  | **41** | **3** | **12** | **1** | **2** | **c.198C>A** | **p.T48N** | **Missense substitution** | **24.5 (48684293; GRCh38-v1.6)** | **Reduced** | **Well** |
|  | 39 | 1.5 | 8 | 5 | 2 | c.272T>C | p.C73R | Missense substitution |  | Reduced | Well |
|  | **77** | **0.2** | **156** | **3** | **2** | **c.278G>C** | **p.V75L** | **Missense substitution** | **25.8 (48684373; GRCh38-v1.6)** | **N.D** | **Well** |
|  | 92 | 0.25 | 5 | 3 | 2 | c.291delC | p.Q80R126X | Missense substitution |  | N.D | Well |
|  | 31 | 5 | 36 | 2 | 2 | c.312G>A | p.R86H | Missense substitution |  | Reduced | Well |
|  | 81 | 8 | 84 | 2 |  |  |  |  |  | N.D | Well |
|  | 29 | 0.5 | 4 | 3 |  |  |  |  |  | Reduced | Well |
|  | 30 | 36 | 84 | 5 |  |  |  |  |  | N.D | Well |
|  | 4 | 9 | 42 | 5 |  |  |  |  |  | N.D | Well |
|  | 47 | 7 | 12 | 4 | 2 | c.345G>A | p.W97X | Nonsense substitution |  | Reduced | Well |
|  | **50** | **1** | **5** | **4** | **3** | **c.367_368insA** | **p.L105Tfs121X** | **Frameshift insertion** | **PT** | **Reduced** | **Well** |
|  | **90** | **5** | **31** | **3** | **4** | **c.446G>T** | **p.E131X** | **Nonsense substitution** | **PT** | **N.D** | **Well** |
|  | 23 | 3 | 15 | 3 | 4 | c.452G>A | p.E133K | Missense substitution |  | Reduced | Well |
|  | 76 | 17 | 30 | 3 |  |  |  |  |  | N.D | Lost to follow up |
|  | **79** | **60** | **96** | **1** | **4** | **c.497_498insA** | **p.R148Kfs168X** | **Frameshift insertion** | **PT** | **N.D** | **Well** |
|  | **17** | **2** | **6** | **4** | **4** | **c.509C>T** | **p.Q152X** | **Nonsense substitution** | **PT** | **Reduced** | **Expired** |
|  | **80** | **1** | **2** | **1** | **5** | **c.536delC** | **p.P161Hfs168X** | **Frameshift deletion** | **PT** | **N.D** | **Well** |
|  | **82** | **0.3** | **24** | **3** | **5** | **c.551delG** | **p.A166Pfs257X** | **Frameshift deletion** | **PT** | **N.D** | **Well** |
|  | **75** | **5** | **7** | **3** | **6** | **c.585_592 delTGCCCCT** | **p.L177Rfs258X** | **Frameshift deletion** | **PT** | **N.D** | **Well** |
|  | 5 | 1 | 84 | 5 | 7 | c.631C>T | p.R211X | Nonsense substitution |  | N.D | Well |
|  | 40 | 8 | 48 | 5 |  |  |  |  |  | Reduced | Expired |
|  | **83** | **4** | **8** | **2** | **7** | **c.720delC** | **p.P222Qfs260X** | **Frameshift deletion** | **PT** | **N.D** | **Well** |
|  | **19** | **1** | **12** | **3** | **7** | **c.721_722insC** | **p.A223Sfs224X** | **Frameshift insertion** | **PT** | **N.D** | **Lost to follow up** |
|  | **85** | **1.5** | **5** | **1** | **9** | **c.968C>T** | **p.Q305X** | **Nonsense substitution** | **PT** | **N.D** | **Lost to follow up** |
|  | 93 | 5 | 9 | 3 | 9 | c.974A>G | p.M307V | Missense substitution |  | N.D | Well |
|  | 94 | 168 | 384 | 5 | 9 | c.974A>G | p.M307V | Missense substitution |  | N.D | Well |
|  | 32 | 1 | 72 | 3 | 10 | c.996delC | p.P314Rfs444X | Frameshift deletion | PT | Absent | Well |
|  | 91 | 36 | 156 | 3 | 10 | c.1016C>T | p.R321X | Nonsense substitution |  | N.D | Well |
|  | 95 | 18 | 28 | 3 |  | c.1016C>T | p.R321X | Nonsense substitution |  |  |  |
|  | **84** | **0.1** | **12** | **3** | **10** | **c.1082delC** | **p.P344Lfs444X** | **Frameshift deletion** | **PT** | **N.D** | **Well** |
|  | 3 | 1.5 | 2.5 | 3 | 10 | c.1086delC | p.P344Lfs444X | Frameshift deletion | PT | N.D | Well |
|  | 43^ | 6 | 204 | 2 | 10 | c.1145C>T | p.R364X | Nonsense substitution |  | Reduced | Well |
|  | 45^' | 1.5 | 3 | 5 |  |  |  |  |  | Reduced | Expired |
|  | **96** | **0** | **1** | **1** | **10** | **c.1179delC** | **p.T375Mfs444X** | **Frameshift deletion** | **PT** | **Reduced** | **Lost to follow up** |
|  | **98** | **0** | **44** | **3** |  |  |  |  |  | **Reduced** | **Lost to follow up** |
|  | **26*~** | **6** | **36** | **3** | **10** | **c.1245delC** | **p.P397Rfs444X** | **Frameshift deletion** | **PT** | **Normal** | **Well** |
|  | **25*~** | **4** | **12** | **5** |  |  |  |  |  | **Reduced** | **Well** |
|  | **46** | **2** | **48** | **4** | **10** | **c.1274_1290delGGGAATGGACCAGCCCC** | **p.GNGPAP407**  **delfs488X** | **Frameshift deletion** | **PT** | **Normal** | **Expired** |
|  | **15** | **2** | **12** | **3** | **12** | **Large deletion** |  | **Large deletion** | **PT** | **N.D** | **Expired** |
|  | **51** | **2** | **18** | **4** | **12** | **c.1564_1567delAGTG** | **p.X503W** | **Frameshift deletion>stop-loss** | **Extension** | **Reduced** | **Expired** |
|  | 47 | 8 | 11 | 5 | 12 | c.1562T>C | p.X503R | Stop-loss |  | Reduced | Expired |
|  | **8** | **2** | **4** | **5** | **IVS3** | **c.415+5G>C** | | **Splice site defect, substitution** | **26.1 (48685638; GRCh38-v1.6)** | **Reduced** | **Well** |
|  | 16 | 0.8 | 9 | 3 | IVS3 | c.416-1G>A | | Splice site defect, substitution |  | N.D | Expired |
|  | **97** | **0** | **36** | **2** | **IVS6** | **c.613+2T>C** | | **Splice site defect, substitution** | **32 (48686136; GRCh38-1.6v)** | **Absent** | **Lost to follow up** |
|  | 89 | 5 | 156 | 5 | IVS6 | c.613+5G>A | | Splice site defect, substitution |  | Normal | Expired |
|  | 20 | 36 | 132 | 3 | IVS7 | c.791-1G>A | | Splice site defect, substitution |  | Reduced | Well |
|  | 21 | 3 | 22 | 3 | IVS8 | c.832+1G>A | | Splice site defect, substitution |  | Reduced | Well |
|  | **9** | **1** | **6** | **5** | **IVS8** | **c.832+3_ +6delGAGT** | | **Splice site defect, substitution** |  | **Reduced** | **Expired** |
|  | 2 | 2 | 54 | 5 | IVS9 | c.985+17G>A | | Splice site defect, substitution |  | N.D | Well |
|  | **24^** | **24** | **72** | **5** | **IVS10** | **c.1393+2, T>G** | | **Splice site defect, substitution** | **33 (48689068; GRCh38-1.6v)** | **Normal** | **Well** |
|  | **42^** | **4** | **192** | **5** |  |  |  |  |  | **Reduced** | **Well** |
|  | **18** | **0** | **9** | **3** | **IVS11** | **c.1507+2T>A** | | **Splice site defect, deletion** | **33 (48689436; GRCh-1.6v)** | **Reduced** | **Expired** |

Variants depicted in bold entries represent novel variants. *Siblings; ^Maternal cousins; ‘Patient had another mutation in *XIAP* gene.

Transcript ID: ENST00000376701.5 for cDNA and protein positions.

**Abbreviations**- PT: Premature termination; CADD Score: Combined Annotation Dependent Depletion (CADD) Scores for Single nucleotide variants; SNV: Single Nucleotide Variants.
