# Supplementary material for: Wiskott Aldrich Syndrome: A Multi-Institutional Experience From India
Source: Front Immunol. 2021 Apr 16;12:627651. doi: 10.3389/fimmu.2021.627651 (PMC8086834; doi:10.3389/fimmu.2021.627651)
Supplement: Supplementary file 5 [file Table_3.docx]

Supplementary table 3: Distribution of genetic variants in XLT and WAS in the study population.

| **Type of mutation** | **XLT**  **(n=11)** | **WAS**  **(n=56)** | **Total**  **(n=67)** | **% out of total (n=67)** |
| --- | --- | --- | --- | --- |
| Nonsense substitution | 2 | 17 | 19 | 28.4 |
| Missense substitution | 4 | 15 | 19 | 28.4 |
| Frameshift deletion | 3 | 10 | 13 | 19.4 |
| Frameshift insertion | 1 | 2 | 3 | 4.5 |
| Splice site defect,  Substitution | 1 | 9 | 10 | 14.9 |
| Splice site defect, deletion |  | 1 | 1 | 1.5 |
| Large deletion |  | 1 | 1 | 1.5 |
| Stop-loss |  | 1 | 1 | 1.5 |
| No. of patients | 11 | 56 | 67 | 100 |
| - Unique variants | 6 (+3) | 38 (+3) | 47 | 70.1 |
| - Novel variants | 5 (+1) | 17* (+1) | 24 | 35.8 |
| - No. of patients with exonic variants | 10 | 46 | 56 | 83.6 |
| - No. of patients with intronic variants | 1 | 10 | 11 | 16.4 |

Variants depicted in brackets were common variants found in XLT as well as WAS. Total WAS with novel variants were 20, two pairs of relative had same variants (i.e., *17 + 1 (common with XLT) + 2 (relatives with same variants) = 20).
