# Supplementary material for: Wiskott Aldrich Syndrome: A Multi-Institutional Experience From India
Source: Front Immunol. 2021 Apr 16;12:627651. doi: 10.3389/fimmu.2021.627651 (PMC8086834; doi:10.3389/fimmu.2021.627651)
Supplement: Supplementary file 6 [file Table_4.docx]

Supplementary table 4**: Type of transplant, conditioning regimen, complications, and outcome of patients who underwent Hematopoietic stem cell transplant**

| **S. No** | **Donor** | **Source of HSCT** | **Conditioning Regimen** | **Engraftment** | | **Acute GVHD** | | **Chimerism** | **Outcome** |
| --- | --- | --- | --- | --- | --- | --- | --- | --- | --- |
|  |  |  |  | **Myeloid** | **Lymphoid** | **Yes/ No** | **Treatment** |  |  |
| 1* | MRD | PBSC | Bleomycin/ Cyclophosphamide | Yes | Yes | No | - | Full | Alive, and well |
| 2* | CB | CB | Fludarabine/ Busulfan/ ATG | Yes | Yes | No | - | Full | Died due to CMV |
| 3* | CB | CB | Fludarabine/ Busulfan/ ATG | Yes | Yes | Yes | - | Full | Died due to GVHD |
| 4* | CB | CB | Fludarabine/ Busulfan | No | No | No | - |  | Died due to pulmonary haemorrhage |
| 5* | MUD | PBSC | Fludarabine/ Busulfan/ ATG | Yes | Yes | No | - | Graft rejection | Died following 2^nd^ HSCT due to Sepsis |
| 6* | Haplo | PBSC | Fludarabine/ Melphalan | Yes | Yes | No | - | Full | Alive, and well |
| 7* | CB | CB | Fludarabine/ Treosulfan/ thiotepa/ ATG | No | No | No | - | - | Died from ARDS prior to engraftment |
| 8* | MRD | PBSC | Fludarabine/ Busulfan | Yes | Yes | Yes | Steroids | Mixed | Alive, and well |
| 9* | Haplo | PBSC | Fludarabine/ Melphalan | Yes | Yes | Yes | Steroids | Mixed | Alive, and well |
| 10* | MUD | PBSC | Fludarabine/ Busulfan/ ATG | Yes | Yes | Yes | Steroids | Full | Alive, and well |
| 11* | MUD | PBSC | Fludarabine/ Busulfan/ ATG | Yes | Yes | Yes | Steroids | Full | Alive, and well |
| 12* | Haplo | PBSC | Fludarabine/ Treosulfan | No | No | No | - |  | Died due to sepsis and graft failure |
| 13* | MRD | PBSC | Fludarabine/ Busulfan | Yes | Yes | Yes | Steroids | Full | Alive, and well |
| 14* | Haplo | PBSC | Fludarabine/ Melphalan | Yes | Yes | No | - | Full | Alive, and well |
| 15* | MRD | PBSC | Fludarabine/ Busulfan | Yes | Yes | Yes | Steroids | Full | Alive, and well |
| 16* | MRD | PBSC | Fludarabine/ Busulfan | Yes | Yes | No | - | Full | Alive, and well |
| 17* | MUD | PBSC | Fludarabine/ Busulfan/ ATG | Yes | Yes | Yes | Steroids | Full | Died due to immune cytopenia |
| 18* | Haplo | PBSC | Fludarabine/ Melphalan | Yes | Yes | No | - | Mixed | Alive, and well |
| 19* | Haplo | PBSC | Fludarabine/ Busulfan | No | No | - | - | Graft failure | Awaiting second HSCT |
| 20* | - | - | - | - | - | No | - | - | Died due to sepsis after transplant |
| 21^#^ | Haplo | PBSC | - | - | - | - | - | Full | Alive, and well |
| 22^¥^ | CB | CB | Busulfan/ Cyclophosphamide/ ATG | No | No | Yes | Steroids | - | Died due to sepsis, ARDS, GVHD |
| 23^¥^ | Haplo | Bone Marrow | Fludarabine/ Busulfan/ ATG | Yes | Yes | No | - | Full | Alive, and well |
| 24^¥^ (36) | Haplo | Bone Marrow | Busulfan/ Cyclophosphamide/ ATG | Yes | Yes | Yes | Steroids, Daclizumab, MMF | Full | Alive, and well |
| 25^$^ | Haplo | - | - | Yes | Yes | - | - | Full | Alive, and well |

Abbreviations: HSCT: Hematopoietic Stem cell transplant; GVHD: Graft versus Host disease; Haplo: Haploidentical; MRD: matched related donor; MUD: Matched unrelated donor; PBSC: Peripheral blood stem cell; CB: Cord blood; ATG: Anti-thymocyte globulin; MMF: Mycophenolate mofetil; ARDS: Acute respiratory distress syndrome.

Treating centre- *Apollo Hospitals, Chennai; # Aster CMI Hospital, Bengaluru; ^¥^ PGIMER, Chandigarh. ^$^KMC, Manipal, Bengaluru
