# Supplementary material for: Wiskott Aldrich Syndrome: A Multi-Institutional Experience From India
Source: Front Immunol. 2021 Apr 16;12:627651. doi: 10.3389/fimmu.2021.627651 (PMC8086834; doi:10.3389/fimmu.2021.627651)
Supplement: Supplementary file 7 [file Table_5.docx]

| **Author** | **Country** | **Number of**  **cases** | **Mean age at diagnosis** | **Eczema** | **Infections** | **Bleeding** | **Autoimmunity** | **Malignancy** |
| --- | --- | --- | --- | --- | --- | --- | --- | --- |
| Sullivan KE et al. (38) 1995 | USA  Canada | 154 | 1.75 months  (0-24.8) | **Total patients with Eczema: 81%** | **Total patients with infections: n/a**  Otitis media: 118/154, 78%  Pneumonia: 68/154, 45%  Diarrhea: 20/154, 13%  Sinusitis: 36/154, 24%  Sepsis: 36/154, 24%  Meningitis: 11/154, 7%  Varicella: 25/154, 16%  EBV: 4/154, 3%; CMV: 4/154, 3%  HSV I or HSV II: 24/154, 6%  Polyomavirus: 2/154, 1%  Molluscum contagiosum: 13/154, 9%; Warts: 10/154, 7%  Yeast/Fungi: 18/154, 12%  Pneumocystis carinii: 13/154, 9%  Other infections:58/154, 38%  Other opportunistic infections: 10/154, 7% | **Total patients with bleeding: 84%**  Petechiae and purpura: 78%  Hematemesis and melena: 28%  Epistaxis, 16%  Oral bleeding: 6%  Intracranial hemorrhage: 2%  Others: 5% (Unusual bleed from umbilical stump; Postsurgical, and post-traumatic bleeding; Testicular hemorrhage; and several subconjunctival hemorrhages) | **Total patients with autoimmunity: 61/ 154, 40%**  AIHA: 22/154, 14%  Vasculitis: 20/154, 13%  Renal disease: 18/154, 12%  Transient arthritis: 17/154, 11%  Chronic arthritis: 15/154, 10%  Schönlein-Henoch purpura: 8/154, 5%  IBD: 5/154, 3%  Dermatomyositis: 1/154, 0.6%  Others: 14/154, 9% (Recurrent angioedema; Neutropenia; Cerebral vasculitis; Uveitis Myositis; Auto-immune hepatitis; Pyoderma gangrenosum; Erythema nodosum; cardiac vasculitis) | **Total patients with malignancy: 21/ 154, 13%**   (Lymphoreticular: 18, and non-lympho-reticular: 3)  Lymphoma: 14/21, 66%  Reticuloendothelial: 1, 4.7%  Glioma: 1, 4.7%  Reticulum cell sarcoma: 1, 4.7%  Lymphoblastic leukemia: 1, 4.7%  Malignant histiocytosis: 1, 4.7%  Testicular carcinoma: 1, 4.7%  Acoustic neuroma: 1, 4.7% |
| Imai et al., (42)  2004 | Japan | 50 | n/a | **Total patients with Eczema: 40/50, 80%**  Transient: 8/50, 16%  Moderate: 20/50, 40%  Severe: 12/50, 24%  (5)  Others:  Asthma: 4/50, 8%  Food allergy: 4/50, 8% | **Total patients with infections: 44/50, 88%**  Details of bacterial infections: n/a  CMV: 5/50, 10%  Candidiasis: 9/50, 18%  Pneumocystis carinii pneumonia: 2/50, 4%  HSV I: 13/115 episodes, 11.3%  Recurrent herpes: 2/50, 4%  Severe varicella: 1/50, 2%  Measles pneumonitis: 2/50, 4%  Mumps meningitis: 2/50, 4%  EBV reactivation: 1/50, 2%  Molluscum contagiosum: 5/50, 10%  Aspergillosis: 3/50, 6% | **Total patients with bleeding: n/a**  ICH: 11/50, 24%  Intestinal bleeding: 18/50, 36% | **Total patients with autoimmunity: 12/50, 24%**  Vasculitis: 4/50, 8%  Arthritis: 3/50, 6%  IBD: 2/50, 4%  AIHA: 3/50, 6%  IgA nephropathy: 5/50, 10%  Chronic renal failure: 2/50, 4% | **Total patients with Malignancy: 5/50, 10%**  Malignant lymphoma: 2/50, 4%  Myelodysplasia: 3/50, 6% |
| Lee et al.  (39) 2009 | China | 35 | 11 months (1- 42 months) | **Total patients with Eczema:**  **35/35, 100%** | **Total patients with infections: n/a**  Pneumonia:13, 37%  Respiratory tract infection: 12/35, 28.5%  Skin abscess: 7/35, 20%  Otitis media: 6/35, 17%  Neisseria meningitidis: 1/35, 2.8%  Streptococcus pneumoniae: 1/35, 2.8%  Herpetic gingivo- stomatitis: 1/35, 2.8%  CMV interstitial pneumonitis: 1/35, 2.8% | **Total patients with bleeding: n/a**  ICH: 2/35, 5.7%  Massive hematemesis: 1/35, 2.8%  Chronic per-rectal bleeding: 19/35, 54.3%  Recurrent epistaxis: 11, 34%  Petechiae: 12/35, 34.3% | **Total patients with autoimmunity: 12/35, 34.2%**  AIHA: 6/35, 17.1%  Neutropenia: 6/35, 17.1% | **Total patients with Malignancy: 1/35, 2.8%**  EBV related high-grade large B-cell lymphoma: 1/35, 2.8% |
| Albert MH et al.  (41)  2010 | France  Japan  United States of America  United Kingdom  Germany  Sweden  Spain  Russia  Israel  Netherlands | 173 cases of XLT | **-** | **-** | **Total patients with infections: 12/173, 6.3%**  Total No. of events: 17  Episodes of Pneumonia: 6/17, 35%  Bacterial meningitis: 4/17, 23.5  Sepsis: 4/17, 23.5  Salmonellosis: 1/17, 5.8%  Orchitis: 1/17, 5.8%  Tuberculosis: 1/17, 5.8% | **Total patients with bleeding: 24, 13.8%**  No. of events: 33  ICH: 18/ 33, 54.5%  Gastrointestinal: 6/ 33, 18.1%  Ear/nose/throat: 4/ 33, 12%  Pulmonary: 2/ 33, 6%  Traumatic, not ICH: 2/ 33, 6%  Retinal: 1/33, 3% | **Total patients with autoimmunity: 21/ 173, 12%**  No. of events: 26  Nephropathy: 9/26, 35%  AIHA: 6/26, 23%  Vasculitis: 3/26, 11.5%  ITP: 4/26, 15%  Arthritis: 3/26, 11.5  Colitis: 1/26, 3.8% | **Total patients with malignancy: 9/ 173, 5.2%**  No. of events: 10  Lymphoma/EBV-LPD: 4/10, 25%  MDS: 1/10, 10%  Spinalioma: 2/10, 20%  Seminoma: 1/10, 10%  ALL: 1/10, 10%  Pancreatic cancer: 1/10  , 10% |
| Shin CR et al., (43) 2012 | Cincinnati, USA | 47 | 1.52 months (0.26-19) | **Total patients with Eczema: 28/47, 59%** | **Total patients with infections: 24/47, 51%** | **Total patients with bleeding: 17, 40%** | **Total patients with autoimmunity: 15/47, 32%** | **Total patients with malignancy: 2, 4.2%** |
| Jin YY  et al.,  (40)  2019 | China | 42 | 5.6 months | **Total patients with Eczema: 29/ 42, 69%**  Drug allergy: 1, 2.3% | **Total patients with infections: 41, 97.6%**  Pneumonia: 23/42, 55%  Diarrhea: 23/42, 55%  Otitis media: 3/42, 7.1%  Thrush: 3/42, 7.1%  Lymphadenitis: 1/42, 2.3%  CMV infection: 1/42, 2.3%  EBV infection: 1/42, 2.3% | **Total patients with bleeding: 38, 90%**  Skin spots or petechial: 27/42, 64.2%  Per-rectal bleeding:21/42, 50%  Epistaxis: 7/42, 16.6%  ICH: 2/42, 4.7%  Multiple-system bleeding: 12/42, 28.5% | **Total patients with autoimmunity: 3/ 42, 7%**  AIHA: 2/42, 4.7%  AIHA with IgA nephropathy: 1/42, 2.3% | **-** |
| Haskologlu et al., (37)  2020 | Turkey | 23 | 24 months (1- 132 months) | **-** | **Total patients with infections: n/a**  Two patients died due to severe infections | **Total patients with bleeding**: 91%  Skin spots or petechia: 91%  **(11 [48%] patients misdiagnosed as ITP initially)** | **Total patients with autoimmunity: 3/ 23, 9%**  Chronic renal failure: 3/23, 9%  (2 with IgA nephropathy, and Leukocytoclastic vasculitis; and 1 with  only leukocytoclastic vasculitis) | **Total patients with malignancy: 1/23, 4.3%**  EBV associated non-Hodgkin’s lymphoma: 1/23, 4.3% |
| Present study | India | 95 | **WAS:** 12 months (5.5- 36)  **XLT:** 51 months (10- 102) | **Total patients with Eczema: 75/95, 79%** | **Total patients with infections: 80/95, 84.2%**  Pneumonia: 52, 54.7%  Otitis Media: 43, 35.7%  CMV: 7, 7.3%; Varicella: 4, 4.2%  MC virus infection: 5, 5.3%  EBV: 2, 2.1% | **Total patients with bleeding: 88/95, 92.6%**  Blood-stained stool: 67, 70.5%  Skin bleed: 54, 56% Epistaxis: 11, 11.5%  Hematuria: 5, 5.3% | **Total patients with autoimmunity: 38/95, 40%**  AIHA: 9, 9.5%  Skin vasculitis: 9, 9.5%  Takayasu arteritis: 1, 1%  Positive antinuclear antibody: 9, 9.5%. | **Total patients with malignancy: 2/95, 2.1%**  NHL: 2, 2.1% |

**Supplementary table 5: Clinical profile in large published cohorts**

**Abbreviations:** EBV: Epstein Barr virus; HSV: Herpes Simplex virus; AIHA: Autoimmune hemolytic anemia; IBD: Inflammatory Bowel Disease; ITP: Immune thrombocytopenic purpura; LPD: Lymphoproliferative Disease; ALL: Acute lymphoid leukemia; CMV: Cytomegalovirus; ICH: Intracranial hemorrhage; MC virus: Molluscum contagiosum; NHL: Non-Hodgkin lymphoma.
